# Early Handling Exerts Anxiolytic Effects and Alters Brain Mitochondrial Dynamics in Adult High Anxiety Mice

**DOI:** 10.1007/s12035-024-04116-5

**Published:** 2024-05-18

**Authors:** Christina Thomou, Markus Nussbaumer, Eleni Grammenou, Chrysoula Komini, Angeliki-Maria Vlaikou, Maria P. Papageorgiou, Michaela D. Filiou

**Affiliations:** 1 Laboratory of Biochemistry, Department of Biological Applications and Technology, University of Ioannina, Ioannina, Greece; 2 Biomedical Research Institute, Foundation for Research and Technology-Hellas (FORTH), Ioannina, Greece; 3 Institute of Biosciences, University of Ioannina, Ioannina, Greece

**Keywords:** Neonatal handling, Post-natal handling, Mitochondria, HAB, Maternal behavior, Early life

## Abstract

Early handling (EH), the brief separation of pups from their mother during early life, has been shown to exert beneficial effects. However, the impact of EH in a high anxiety background as well as the role of brain mitochondria in shaping EH-driven responses remain elusive.

Here, we used a high (HAB) vs. normal (NAB) anxiety-related behavior mouse model to study how EH affects pup and dam behavior in divergent anxiety backgrounds. We also investigated EH-induced effects at the protein and mRNA levels in adult male HAB mice in the hypothalamus, the prefrontal cortex, and the hippocampus by examining the same mitochondrial/energy pathways and mitochondrial dynamics mechanisms (fission, fusion, biogenesis, and mitophagy) in all three brain regions.

EH exerts anxiolytic effects in adult HAB but not NAB male mice and does not affect HAB or NAB maternal behavior, although basal HAB vs. NAB maternal behaviors differ. In adult HAB male mice, EH does not impact oxidative phosphorylation (OXPHOS) and oxidative stress in any of the brain regions studied but leads to increased protein expression of glycolysis enzymes and a correlation of anxiety-related behavior with Krebs cycle enzymes in HAB mice in the hypothalamus. Intriguingly, EH alters mitochondrial dynamics by increasing hypothalamic DRP1, OPA1, and PGC1a protein levels. At the mRNA level, we observe altered, EH-driven mitochondrial dynamics mRNA signatures which predominantly affect the prefrontal cortex.

Taken together, our results show that EH exerts anxiolytic effects in adulthood in high anxiety and modulates mitochondrial dynamics pathways in a brain region-specific manner.

**Supplementary Information:**

The online version contains supplementary material available at 10.1007/s12035-024-04116-5.

## Introduction

Early life experiences are crucial for shaping adult life outcomes [1, 2]. Early handling (EH) or neonatal handling, the brief and repeated separation of the pups from their mother during their first days after birth, is an early life intervention which has been shown to confer adaptive behavior in later life and thus enhance the ability of stress-coping, as opposed to a stimulus-poor environment [3, 4]. EH has been predominantly studied in rats; in Wistar rats, EH resulted in decreased anxiety-related behavior in the elevated plus maze (EPM) in adult males and females [5, 6] and in increased exploratory behavior in the open field test (OFT) in adult males [7]. In few studies performed in mice, EH decreased anxiety-related behavior in the EPM in adult male NMRI mice [8] but showed no effect on anxiety-related behavior in the EPM in adult male C57BL/6 mice [9]. In another study, adult C57BL/6, but not DBA/2 mice, showed a trend towards decreased anxiety-related behavior in the elevated zero maze [10].

In addition to the heterogeneity of EH effects on anxiety-related behavior in different mouse strains, the impact of EH on maternal and pup behavior in a high anxiety background remains unexplored. To address the behavioral effects of EH in highly anxious dams and adult offspring, we used a well-characterized mouse model of high trait anxiety based on a CD1 background and generated by selective inbreeding according to mouse behavior in the EPM [11]. Mice spending most of their time in the EPM closed arms gave rise to the high anxiety-related behavior (HAB) line and are compared to their normal anxiety-related behavior (NAB) counterparts. Mice spending more time in the EPM open arms gave rise to the low anxiety-related behavior (LAB) line [12, 13]. At the molecular level, EH has been shown to alter monoamine as well as NMDA and serotonin receptor levels in a brain region- and sex-dependent manner [14–16], increase the density of calcium binding proteins in the hippocampus and lateral amygdala [17], increase the mu-opioid receptor protein levels in multiple brain regions [5], and decrease plasma corticosterone levels [18] in rats. EH also resulted in decreased mRNA levels of serotonin receptors in early life, and conversely, increased mRNA levels of serotonin receptors in adulthood in the frontal cortex of male C57BL/6 mice [9].

Despite the availability of studies addressing the underlying molecular mechanisms of EH, little is known about EH-driven effects on mitochondrial function in the adult brain. Accumulating evidence deriving from molecular characterization [19, 20] and psychiatric drug action [21–24] studies in animal models of psychopathology has highlighted the role of mitochondria in the pathophysiology of major psychiatric disorders. Emerging preclinical and clinical work points towards a bidirectional link between high anxiety and brain mitochondria [25]. Recently, mitochondrial dynamics, the orchestrated interplay of mitochondrial fission, fusion, biogenesis, and mitophagy for mitochondria quality control [26], has been shown to regulate anxiety-related behaviors in rats [27] and mice [28]. In HAB mice, we have previously found altered brain mitochondrial pathways, including mitochondrial metabolism, mitochondrial import/transport, and oxidative stress [29–32].

In this work, we investigated the impact of EH in pup and dam behavior in a high anxiety background by comparing HAB vs. NAB mice and analyzed the mitochondrial correlates of the EH-driven behavioral effects in adult HAB mice. First, we assessed how EH affects sociability, anxiety-related, and depressive-like behavior in male HAB and NAB mice by an elaborate behavioral testing battery. Additionally, we investigated the effects of EH in HAB and NAB dams by observing maternal behavior during the EH protocol. We then looked for persistent molecular effects of EH in adulthood by assessing a series of mitochondrial parameters, including oxidative phosphorylation (OXPHOS), oxidative stress, metabolism, and mitochondrial dynamics in three brain regions in HAB male mice. These brain regions, namely the hypothalamus [33], the prefrontal cortex [34], and the hippocampus [35], were selected due to their involvement in anxiety circuits. Our results show that EH exerts anxiolytic effects in HAB male mice, which are mediated by alterations in the brain mitochondrial dynamics machinery, predominantly in the hypothalamus.

## Materials and Methods

### Animals

Mice from both HAB and NAB lines were mated. All breeders had been screened at 8 weeks of age on the EPM test to verify their anxiety phenotype. As soon as pregnancy was detected, males were removed from the breeding cages, and pregnant females were left undisturbed until birth (post-natal day (PND) 0 for pups). All animals were housed in type 3 macrolone cages and kept under standard conditions (12 h light/12 h dark cycle, lights on at 6:30 a.m., temperature 21–25 °C, humidity 60%, tap water and food ad libitum) in the animal facility of the University of Ioannina. Mouse experiments were approved by the local authorities and conducted in accordance with the European Communities Council Directives 2010/63/EU.

### Early Handling (EH) Paradigm

On PND 0, HAB and NAB pups were culled up to 8–10 per litter (in total, 11 ΗΑΒ and 8 NAB litters), and each cage was randomly assigned to early handling (EH) and no handling (NH) litters. HAB EH (*n* = 5 litters) and NAB EH (*n* = 4 litters) pups were removed from their home cage for 15 min per day, from PND 1 to PND 14 [8]. During separation time, all pups were placed in small boxes with bedding and kept in a different room. After the EH intervention, pups were returned to their home cage. Throughout the EH protocol, pup handling took place at 3:30 p.m. (lights off at 6:30 p.m.). HAB NH (*n* = 6 litters) and NAB NH (*n* = 4 litters) pups were only handled from PND 8 on and once per week for cage change (animal facility rearing condition). Weekly cage change was also done in EH cages. On PND 28, all pups were weaned into cages of 3–6 same-sex littermates per group. For subsequent behavioral and molecular analyses in this study, we only used male mice.

### Maternal Observations

Maternal behavior of HAB EH (*n* = 5), HAB NH (*n* = 6), NAB EH (*n* = 4), and NAB NH (*n* = 4) dams was observed during the EH paradigm from PND 2 to PND 7 in their home cages under undisturbed conditions. The following behaviors were observed (listed from more to less active care behaviors and described in detail in Table S1): licking-grooming, arched-back nursing, nursing, nest building, self-maintenance, exploration, and inactivity (from [36] and [37] with modifications). Each day, the behavior of each dam was observed in five sessions at the following time points: 5:30 p.m., 7:30 p.m., 9:30 p.m., 8:30 a.m., and 11:30 a.m. The time of the first session (5:30 p.m.) was chosen to allow for habituation of the EH dams after handling procedure. For the dark phase observations, dim red light was used. In each session, dam behavior was observed for a duration of 60 min, with a sampling interval of 2 min, resulting in a total of 30 observations per session, 150 observations per day, and 900 observations for the entire duration of the observation protocol (PND 2–7). At the start of each session, the type of behavior of the dam was recorded (focal sampling) with instantaneous recording. Analysis of maternal behavior was performed for the whole 6-day observation period (PND 2–7), for 3-day periods (PND 2–4 and PND 5–7), and for each day within the total observation period. The percentage of a given behavior per day per dam was calculated as follows: (number of observations of the given behavior/150) × 100.

### Pup Behavioral Characterization

**Fig. 1:**
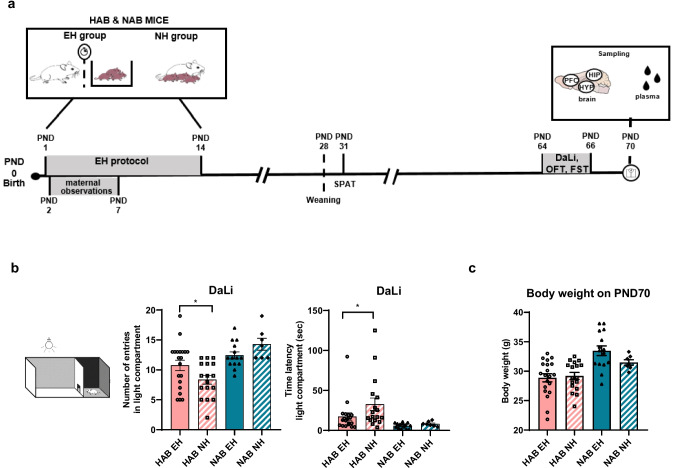
Early handling (EH) induces anxiolytic effects in HAB EH male mice. **a** EH paradigm experimental setup. EH was applied to the pups from PND 1 to PND 14. SPAT was performed on PND 31, followed by DaLi (PND 64), OFT (PND 65), FST (PND 66), and sampling on PND 70. **b** HAB EH male mice enter more times in the DaLi light compartment (*p = 0.0240; HAB EH *n* = 20, HAB NH *n* = 17, NAB EH *n* = 14, and NAB NH *n* = 7) and show decreased latency to the first entry in the DaLi light compartment (*p = 0.0366; HAB EH *n* = 19, HAB NH *n* = 17, NAB EH *n* = 14, and NAB NH *n* = 7) compared to HAB NH mice. No EH-induced anxiolytic effects are observed in NAB male mice. **c** EH does not affect body weight on PND 70 in male HAB and NAB mice (HAB EH *n* = 20, HAB NH *n* = 17, NAB EH *n* = 15, and NAB NH *n* = 7). EH, early handling; NH, no handling; HAB EH, early handling HAB; HAB NH, no handling HAB; NAB EH, early handing NAB; NAB NH, no handling NAB; PND, post-natal day; HYP, hypothalamus; PFC, prefrontal cortex; HIP, hippocampus

Pups were subjected to the social preference-avoidance test (SPAT) on PND 31. Pups were tested on the dark–light box (DaLi) test on PND 64, followed by OFT on PND 65 and forced swim test (FST) on PND 66 (Fig. 1a). Behavioral testing was performed in a randomized cage order, by the same experimenter blind to the treatment for all animals, according to established protocols [38, 39]. Behavioral tests were video-recorded and then scored using the BehaView Software (ver.0.0.16) by an experimenter blind to the treatment.

#### Social Preference-Avoidance Test (SPAT)

The test consisted of 2 sessions of 150 s each with an intersession interval of approximately 30 s. The test was performed in an open field (45 cm × 30 cm × 45 cm). During the first session, the animal was presented with an empty wire grid cup (non-social stimulus) and left to explore. After the intersession interval, the animal was again placed in the apparatus and presented with an unfamiliar same-sex conspecific in the wire grid cup (social stimulus). In both sessions, the number of interactions and total time of interactions with non-social and social stimulus were recorded. The interaction area was defined as 2 cm from the wire grid cup with the animal facing the non-social/social stimulus. Additionally, the social preference-avoidance (SPA) index was calculated as follows: interaction time with social [stimulus/(interaction time with social stimulus + interaction time with non-social stimulus)] × 100. A SPA index of < 50% is considered social avoidance, 50% constitutes a lack of social preference, whereas > 50% is considered social preference [40]. Mice that did not investigate non-social and/or social stimulus were excluded from further analysis. One HAB EH pup was lost after the SPAT and was not included in subsequent analyses.

#### Dark–Light Box (DaLi)

The light compartment was illuminated with 400 lx and the dark compartment with 5 lx. The animal was placed in the apparatus and left to explore for 5 min. The starting compartment was the dark compartment with the head of the animal facing away from the aperture. The time spent in the light compartment, number of entries to the light compartment, and latency to the first entry to the light compartment were recorded. One ΝAB NH animal climbed out of the apparatus and was subsequently excluded from the analysis.

#### Open Field Test (OFT)

Mice were introduced to the apparatus (60 cm × 60 cm × 40 cm), with the head facing towards a corner and left to explore for 5 min. The recorded video was overlaid with horizontal and vertical lines which form a grid of 12 outer and 4 inner boxes (center area). Transitions between grid boxes (line crossings), time spent in, entries to, and latency to the first entry to the inner zone were recorded for the total test duration.

#### Forced Swim Test (FST)

FST was performed as previously described [41]. Mice were placed in a beaker with water (~ 20–22 °C) from which they could not escape. The duration of the test was 6 min. Struggling, swimming, and floating behaviors as well as latency to the first floating event were recorded. After completion of the test, the animal was briefly dried with a cloth towel.

### Animal Sampling

On PND 70, pups were weighed and sacrificed by anesthetization with isoflurane followed by rapid decapitation. The prefrontal cortex, hypothalamus, and hippocampus were dissected according to the mouse brain atlas [42], snap-frozen in liquid nitrogen, and stored at − 80 °C. Plasma was collected from trunk blood by centrifugation at 1300 g for 10 min at 4 °C. For molecular analysis, HAB EH (*n* = 20) vs. HAB NH (*n* = 17) male mice were compared, a subgroup of which was used for protein and biochemical assays (Western blots/total antioxidant capacity (TAC)/carbonylation) (11 HAB EH vs. 10 HAB NH mice) and a subgroup for mitochondrial dynamics mRNA analysis (9 HAB EH vs. 7 HAB NH mice).

### Homogenization/Protein Extraction

Hypothalamus, prefrontal cortex, and hippocampus samples selected for Western blots/TAC/carbonylation were homogenized in RIPA lysis solution (1:10 w/v, 150 mM, 1% NP-40, 0.5% sodium deoxycholate, 0.1% SDS, 50 mM Tris-HCl pH = 8) with a 1:100 (v/v) protease/phosphatase inhibitor cocktail (Sigma-Aldrich, Darmstadt, Germany) followed by sonication using a Branson Digital Sonifier (Marshall Scientific, Hampton, NH, USA) for 10 s at 35% amplitude. After centrifugation at 10,000 g for 10 min at 4 °C, the supernatant was collected, and samples were used for further biochemical assays.

### Western Blot

For all Western blots, the same 11 HAB EH and 10 HAB NH animals were used. Protein content of each sample was estimated by Bradford assay. Western blot was performed as previously described [43] with modifications. Briefly, for each antibody and brain region, samples were loaded on a 26-well pre-cast gel (Bio-Rad, Hercules, CA, USA). Prior to loading, all samples were denatured in a heat block (Thermomixer Comfort, Eppendorf, Hamburg, Germany) for 4 min at 99 °C apart from the samples used for Mitoprofile immunodetection (Table S2) which were not denatured, according to manufacturer’s instructions. Per sample, 15 µg of protein were loaded and separated via 12% SDS-PAGE using a Criterion™ Cell gel electrophoresis chamber (Bio-Rad) and transferred onto a nitrocellulose membrane (GE Healthcare Life Science, Chicago, IL) using a Criterion™ Blotter (Bio-Rad) or a semi-dry Trans-Blot Turbo transfer system (Bio-Rad). Membranes were blocked for 1.5 h in room temperature with a blocking buffer of 5% (w/v) Carnation instant nonfat dry milk in Tris-Buffered Saline with 0.1% Tween 20 (TBS-T) and incubated overnight with primary antibodies followed by incubation with a 1:5000 anti-mouse or 1:10,000 anti-rabbit secondary antibody for 1.5 h in room temperature. Antibody details and dilutions are provided in Table S2. Protein signals were detected with ECL chemiluminescence Forte (Millipore, Bedford, MA, USA) and visualized using a ChemiDoc XRS + System (Bio-Rad). Band signal intensity was quantified using the Image Lab Software (v. 5.0, BioRad). Equal total protein loading was ensured by Coomassie gel staining, Ponceau membrane staining, and signal intensity comparison using ImageJ 1.53t (Wayne Rasband, NIH, Bethesda, MD, USA), similarly to established protocols [29, 39].

### Total Antioxidant Capacity (TAC) Assay

TAC in the prefrontal cortex, hippocampus, and plasma was measured as previously described [44, 45]. Briefly, all samples were incubated with a pre-heated reagent solution (0.1 M phosphate buffer pH = 8.3, 100% ethanol, 100 mM EDTA, 0.5 g/5 ml Thesit, 5 mg/500 µl dichlorophenolindophenol (DCIP), H_2_Ο for injection) in a microplate. For brain samples, 30 µl of tissue homogenate was diluted with 20 µl of H_2_O for injection. For plasma, 50 µl was used. A 0.1 mM reduced glutathione solution (2 mM reduced glutathione pH = 6.5, 50 mM phosphate buffer, 1 mM EDTA) was used as a calibrator. Absorbance was measured in a microplate reader (UT2100C, MRC Ltd, Holon, Israel) at *λ* = 630 nm at 20 s and 120 s after adding the reagent solution. Quantification was performed as previously described [45]. For the brain homogenates, TAC results were normalized based on sample protein content.

### Carbonylation Assay

The carbonylation assay was performed as previously described [46, 47]. For each sample, a corresponding blank sample was prepared. Per sample, a volume corresponding to 1.2 mg/ml protein was used, filled by dH_2_O up to 500 µl. 2,4-Dinitrophenylhydrazine solution was added to the target samples and 2 M HCl to the blank samples, respectively, followed by vortexing every 10 min for 1 h. Thirty percent trichloroacetic acid was added to all samples which were then vortexed and incubated on ice for 10 min. After centrifugation at 11,000 g for 15 min at room temperature, the supernatant was discarded, and 1:1 (v/v) ethanol/ethyl acetate was added to the pellet followed by vortexing and a 10 min incubation at room temperature. All samples were then centrifuged at 11,000 g for 15 min at room temperature. The washing step, including the addition of the 1:1 (v/v) ethanol/ethyl acetate, the incubation, and the centrifugation, was repeated twice. Finally, 6 M guanidine hydrochloride, 20 mM potassium dihydrogen pH = 2.3 solution was added to the pellets, and samples were incubated at 37 °C for 45 min. Absorbance was measured at *λ* = 380 nm (Spectro UV-11, MRC Ltd), and the amount of carbonylated proteins was estimated as previously described [46, 47].

### RNA Extraction

HAB EH (*n* = 9) and HAB NH (*n* = 7) samples from the hypothalamus, prefrontal cortex, and hippocampus were used for RNA extraction. RNA extraction was performed by the NucleoSpin® RNA kit (Macherey–Nagel, Düren, Germany) according to the manufacturer’s instructions. RNA sample quantity was measured with NanoDrop (Thermo Fischer Scientific, Waltham, MA, USA).

### Quantitative Real-Time PCR (qRT-PCR)

For the qRT-PCR polymerization reaction, the SYBR fast one-step qRT-PCR Universal Kit (Kapa Biosystems, Potters Bar, UK) was utilized according to the manufacturer’s instructions. Per reaction, 20 ng of the extracted RNA from each sample was used. The reactions were run in a CFX Connect™ Optics Module thermocycler (BioRad). Signal recognition particle 14 (*Srp14*) was used as a reference gene. All data acquired from qRT-PCRs were analyzed with the 2^−ΔΔCT^ method [48]. All primer sequences are listed in Table S3.

### Statistical Approach

Data were analyzed by GraphPad Prism 8 (GraphPad Software, San Diego, CA, USA). Normality was assessed by the Shapiro–Wilk test. *p* < 0.05 was considered statistically significant, and 0.05 ≤ *p* < 0.1 a trend. Unless otherwise specified, data are presented as mean with ± SEM.

#### Dam Behavioral Analysis

For maternal observations assessing the effect of EH in HAB and NAB mice, repeated 2-way ANOVAs (main effects: *time*, *line*, *treatment*) were used. To compare basal HAB NH and NAB NH dams, repeated 2-way ANOVAs (main effects: *time*, *line*) were used. Only *line* and *treatment* effects (but not time effects) are discussed. Upon a significant (or trend towards) main effect or interaction, analyses were followed by *post-hoc* 2-group comparisons.

#### Pup Behavioral Analysis

For pup behavioral/body weight data, 2-way ANOVAs were performed to investigate main effects (*line*, *treatment*) and their interaction. Upon a significant main effect or interaction, analyses were followed by *post-hoc* 2-group comparisons. For pup data, only differences between HAB ΕΗ vs. HAB NH and NAB EH vs. NAB NH mice are reported. Comparisons between HAB EH vs. NAB EH and between HAB NH vs. NAB NH mice are not discussed.

#### Molecular Analysis

To compare molecular outcomes in HAB EH vs. HAB NH male mice, the unpaired *t*-test (two-tailed) with Welch’s correction assuming unequal standard deviations was used for normal distributions, and the Mann–Whitney non-parametric test (two-tailed) was used for non-normal distributions.

#### Correlation Analysis of Behavioral and Molecular Data

Correlations were performed for the DaLi test parameters which were found altered between HAB EH vs. HAB NH male mice (i.e., entries in light compartment, latency to the first entry in light compartment) and the molecular data of each brain region (Western blot, qRT-PCR, ΤΑC, carbonylation). For normally distributed data and non-normally distributed data, the Pearson and Spearman correlations were used, respectively.

## Results

### Early Handling (EH) Exerts Anxiolytic Effects in HAB but Not in NAB Male Mice in Adulthood

HAB and NAB pups were exposed to an EH protocol (Fig. 1a), and the persistent effects of this early life intervention were assessed in male HAB and NAB mice. For SPAT on PND 31, we observed a significant *line* effect in total interaction time (****p < 0.0001, *F* ratio (1, 46) = 24.57) and a trend for a *treatment* effect (*p* = 0.0734 (T), *F* ratio (1, 46) = 3.358) in SPA index. *Post-hoc* comparisons showed that HAB EH has a lower SPA index compared to HAB NH male mice (*p = 0.0110) (Fig. S1a), indicating that EH decreases sociability in HAB but not in NAB mice.

In adulthood, EH exerted anxiolytic effects in HAB mice. In DaLi, there was a significant interaction *treatment* × *line* effect for the number of entries in the light compartment (*p = 0.0203, *F* ratio (1, 54) = 5.723) and significant *line* effects for entries in the light compartment (****p < 0.0001, *F* ratio (1, 54) = 18.32), latency to the first entry in the light compartment (**p = 0.0048, *F* ratio (1, 53) = 8.67) and time spent in the light compartment (****p < 0.0001, *F* ratio (1, 54) = 109.00) (Figs. 1b and S1b). *Post-hoc* comparisons showed that HAB EH mice enter more times in the light compartment and have decreased latency to the first entry in the light compartment compared to HAB NH mice. This EH-driven anxiolytic effect in DaLi was specific for HAB male mice as EH exerted no anxiolytic effects in NAB male mice (Fig. 1b) and was not dependent on litter effects (Fig. S1c**)**.

In the OFT, significant *line* effects were observed for all parameters assessed, namely time in the center (***p = 0.0002, *F* ratio (1, 55) = 15.52), entries in the center (****p < 0.0001, *F* ratio (1, 55) = 36.76), latency to the first entry in the center (***p = 0.0099, *F* ratio (1, 55) = 7.145), and line crossings (****p < 0.0001, *F* ratio (1, 55) = 51.70). *Post-hoc* comparisons showed no effects of EH in HAB or in NAB male mice in locomotion, which was measured by line crossings, and in all other OFT parameters examined (Fig. S1d). For depressive-like behavior assessed by the FST, there was a significant *line* effect for floating time (****p < 0.0001, *F* ratio (1, 55) = 26.75). *Post-hoc* comparisons showed no ΕΗ-induced effects on depressive-like behavior in HAB or NAB mice, with the exception of a trend in latency towards the first floating event in HAB EH vs. HAB NH mice (*p* = 0.0566 (T)) (Fig. S1e). EH did not result in body weight differences in adult HAB or NAB mice on sampling day (PND 70) (Fig. 1c).

### Early Handling (EH) Does Not Affect Maternal Behavior

**Fig. 2:**
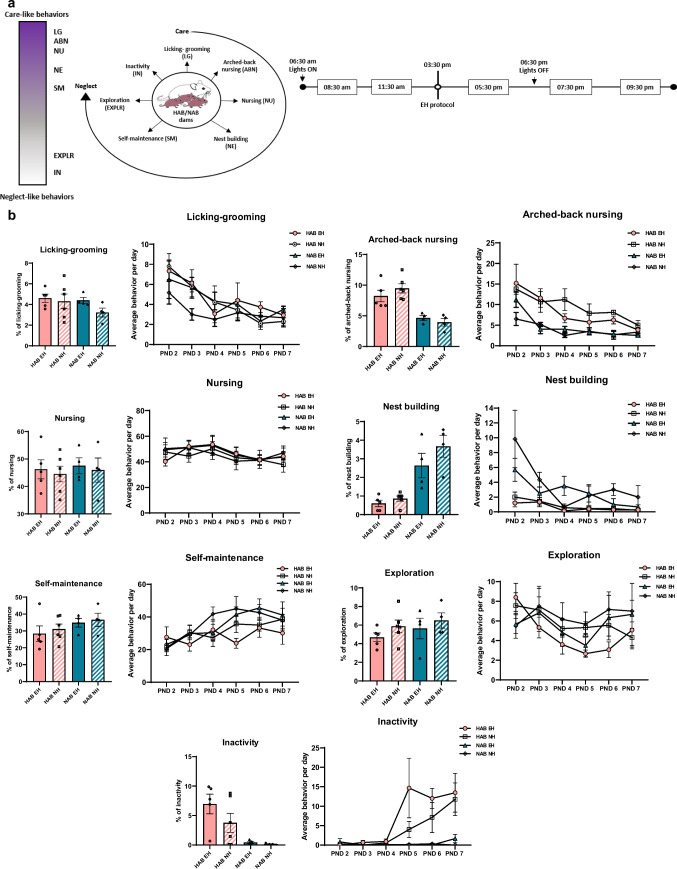
Early handling (EH) does not affect maternal care behavior in HAB and NAB dams. **a** Experimental setup for maternal observations. Maternal behaviors are presented from more (licking-grooming, arched-back nursing, nursing, nest building) towards less (self-maintenance, exploration, inactivity) active care of the pups. Maternal observations were collected from PND 2 to PND 7 on predetermined time points per day. **b** EH does not affect maternal behavior in HAB EH (*n* = 5) vs. HAB NH (*n* = 6) and in NAB EH (*n* = 4) vs. NAB NH (*n* = 4) dams, as shown by the time course analysis of daily assessment of maternal observations. Here, we only discuss differences between HAB EH vs. HAB NH and NAB EH vs. NAB NH dams. EH, early handling; HAB EH, early handling HAB; HAB NH, no handling HAB; NAB EH, early handing NAB; NAB NH, no handling NAB; PND, post-natal day

As the dam-pup bond is briefly and transiently disrupted during EH, we then asked whether EH affects maternal behavior of HAB and NAB dams. We assessed different maternal behaviors, ranging from increased to decreased maternal care (described in detail in Table S1) from PND 2 to PND 7 in defined time points per day during the EH protocol (Fig. 2a). Overall, EH does not substantially impact maternal behavior in HAB and NAB dams. Repeated 2-way ANOVA measures for the whole 6-day observation period, and for the 3-day intervals of maternal observations (PND 2–4 and PND 5–7), showed no effect of EH in HAB and NAB dams in all maternal behaviors assessed, including licking-grooming, arched back nursing, nursing, nest building, self-maintenance, exploration, and inactivity (Fig. 2b).

### Basal HAB and NAB Maternal Behavior Differs

**Fig. 3:**
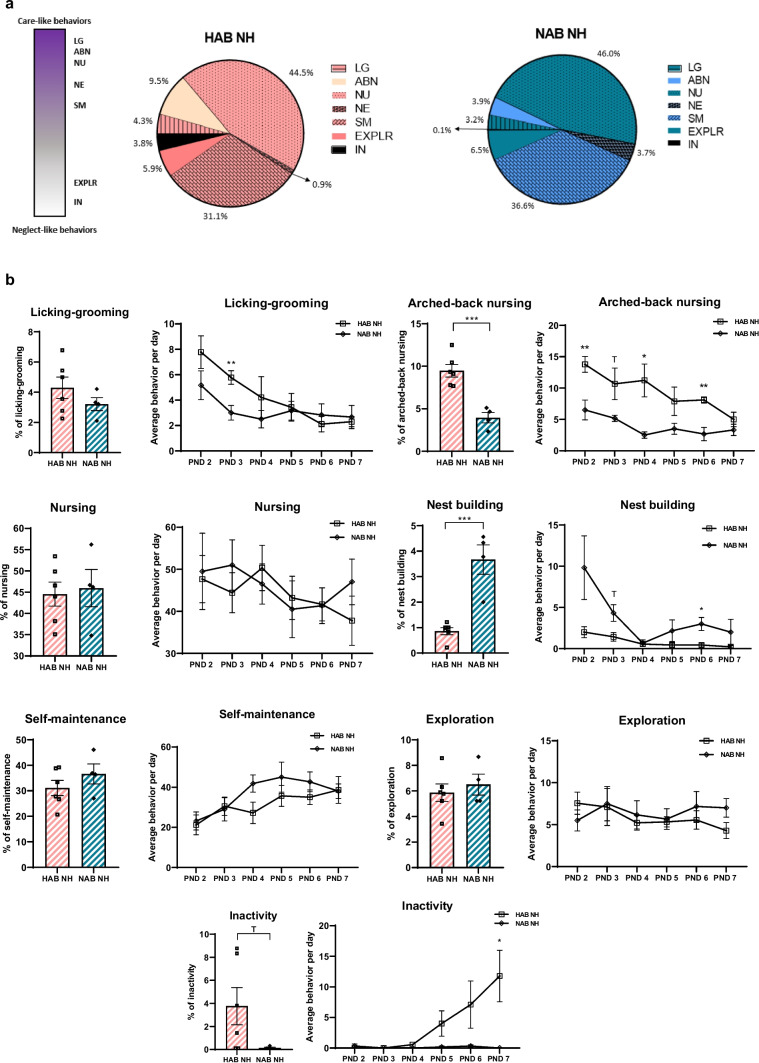
Divergent basal maternal behavior in HAB and NAB dams. **a** Pie charts for the maternal observations for HAB NH and NAB NH in %. Maternal behaviors are presented from more active (licking-grooming, arched-back nursing, nursing, nest building) towards less active (self-maintenance, exploration, inactivity) care of the pups. Data are presented as the average % per behavior for the 6-day observation period. **b** Maternal behavior summary for the 6-day observation period and time course analysis of behaviors for HAB NH (*n* = 6) vs. NAB NH (*n* = 4) dams. Increased arched-back nursing on PND 2 (**p = 0.0098), PND 3 (*p* = 0.0818 (T), PND 4 (*p = 0.0200), and PND 6 (**p = 0.0077) and licking-grooming on PND 3 (**p = 0.0093) were observed in HAB NH compared to NAB NH dams. HAB NH dams show decreased nest building behavior on PND 3 (*p* = 0.0544 (T)) and PND 6 (*p = 0.0439) and increased inactivity on PND 7 (*p = 0.0378) compared to NAB NH dams. Significant *line* effects for the 6-day observation period are noted on the bar graphs. EH, early handling; HAB NH, no handling HAB; NAB NH, no handling NAB; PND, post-natal day; LG, licking-grooming; ABN, arched-back nursing; NU, nursing; NE, nest building; SM, self-maintenance; EXPLR, exploration; IN, inactivity; T, trend

HAB and NAB mice differ in a range of behaviors [11]. Therefore, we investigated whether HAB and NAB dams differ in their basal maternal behavior as well, by comparing HAB NH vs. NAB NH dams. We observed a divergent distribution between HAB and NAB maternal behavior patterns, with HAB dams spending more % time (15.4%) in active care behaviors (licking-grooming and arched-back nursing) compared to NAB dams (7.1%) (Fig. 3a). Time course analysis of maternal behavior for the whole observation period (PND 2–7) showed a significant *line* effect for arched-back nursing (***p = 0.0005, *F* ratio (1, 8) = 31.13). Furthermore, we found a *line* × *time* interaction (*p = 0.0348, *F* ratio (5, 40) = 2.686) and a *line* effect in nest building behavior (***p = 0.0004, *F* ratio (1, 8) = 32.99). For inactive behavior, there is a significant *line* × *time* interaction (*p = 0.0107, *F* ratio (5, 40) = 3.465) and a trend for a *line* effect (*p* = 0.0999 (T), *F* ratio (1, 8) = 3.459) (Fig. 3b).

When analyzing the 3-day intervals, for PND 2–4, we observed a trend for a *line* effect in licking-grooming of the pups (*p* = 0.0906 (T), *F* ratio (1, 8) = 3.701). Moreover, we found a significant *line* effect for arched-back nursing (***p = 0.0001, *F* ratio (1, 8) = 47.58). For nest building behavior, there is a significant interaction between *line* × *time* (*p = 0.0487, *F* ratio (2, 16) = 3.671) as well as a *line* effect (**p = 0.0062, *F* ratio (1, 8) = 13.52). For PND 5–7, we found a significant *line* effect for arched-back nursing (**p = 0.0097, *F* ratio (1, 8) = 11.39) as well as a trend for a *line* effect for nest building behavior (*p* = 0.0544 (T), *F* ratio (1, 8) = 5.073). For inactivity, there are trends for an interaction between *line* × *time* (*p* = 0.0690 (T), *F* ratio (2, 16) = 3.175) and a *line* effect (*p* = 0.0970 (T), *F* ratio (1, 8) = 3.532).

*Post-hoc* 2-group comparisons revealed that HAB NH dams show increased arched-back nursing behavior on PND 2, PND 3 (trend), PND 4, and PND 6 as well as increased licking-grooming behavior on PND 3 compared to NAB NH dams (Fig. 3b). These data further indicate that HAB dams spend more time caring for their pups compared to NAB counterparts. *Post-hoc* comparisons also showed decreased nest building behavior on PND 3 (trend) and PND 6 and increased inactivity on PND 7 in HAB NH compared to NAB NH dams (Fig. 3b).

### Early Handling (EH) Does Not Affect Oxidative Phosphorylation (OXPHOS) and Oxidative Stress in HAB Male Mice

**Fig. 4:**
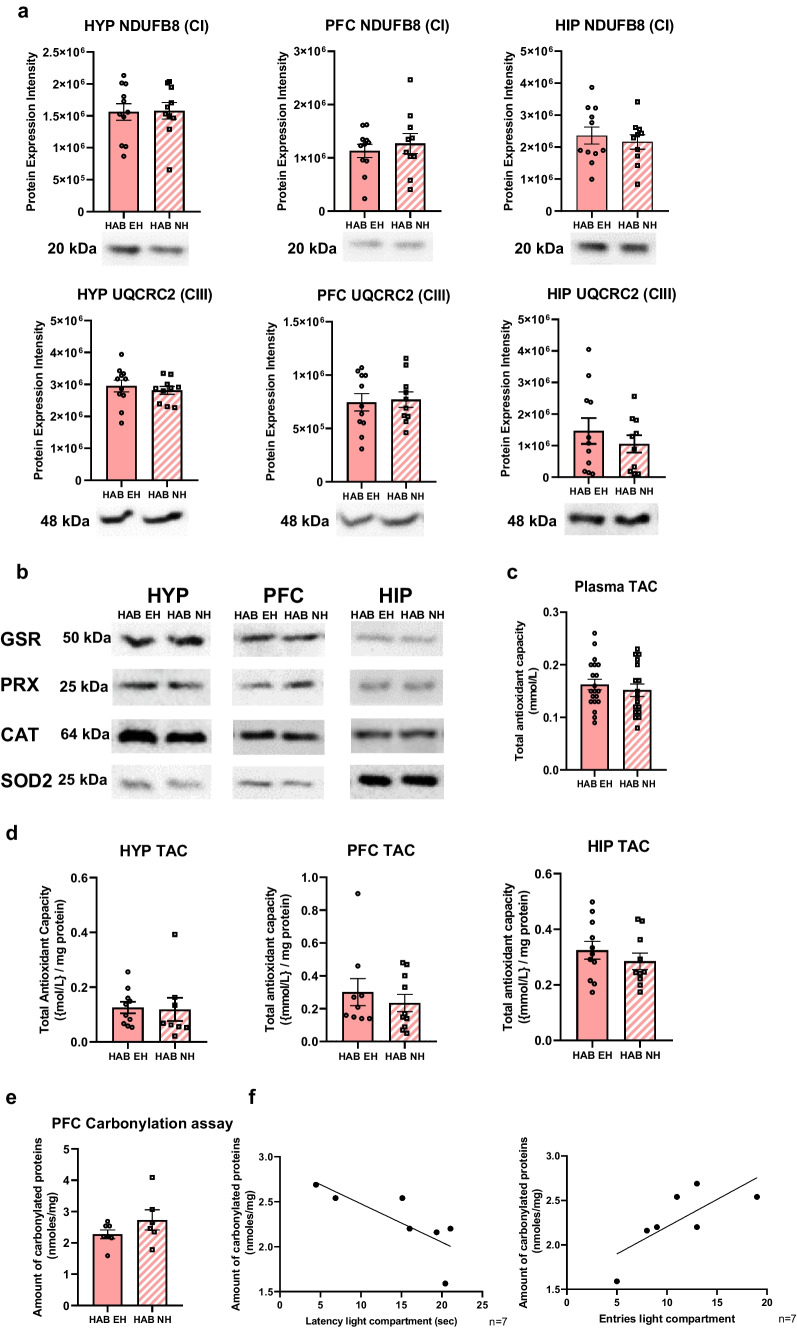
No effects of early handling (EH) on OXPHOS, oxidative stress, and metabolism in adult HAB male mice. **a** Representative data of OXPHOS complex I NDUFB8 and complex III UQCRC2 proteins in hypothalamus, prefrontal cortex, and hippocampus indicating no expression differences in HAB EH (*n* = 11) vs. HAB NH (*n* = 10) mice. **b** Representative Western blots of GSR, PRX, CAT, and SOD2 in hypothalamus, prefrontal cortex, and hippocampus, indicating no expression differences between HAB EH and HAB NH male mice (for quantification data see Fig. S2). **c** No differences in TAC between HAB EH (*n* = 20) and HAB NH (*n* = 17) plasma. **d** No differences in TAC between HAB EH and HAB NH male mice in hypothalamus (HAB EH *n* = 10, HAB NH *n* = 8), prefrontal cortex (HAB EH *n* = 9, HAB NH *n* = 10), and hippocampus (HAB EH *n* = 11, HAB NH *n* = 10). **e** No differences in the amount of carbonylated proteins per mg protein content between HAB EH (*n* = 7) and HAB NH (*n* = 6) mice in the prefrontal cortex. **f** The amount of carbonylated proteins negatively correlates with the latency to the first entry in the DaLi light compartment for HAB EH mice (Pearson *r* =  − 0.7724, *p = 0.0418) and shows a trend towards positive correlation with the number of entries to the light compartment (Pearson *r* = 0.7502, *p* = 0.0521 (T)) in HAB EH mice. Full Western blot data and loading control quantifications are provided in Figs. S3 and S4, respectively. HAB EH, early handling HAB; HAB NH, no handling HAB; HYP, hypothalamus; PFC, prefrontal cortex; HIP, hippocampus; CI, OXPHOS complex I; CIII, OXPHOS complex III; T, trend

Since the anxiolytic effects of EH were specific for HAB mice, our molecular analyses focused on HAB EH vs. HAB NH male mice to elucidate EH-driven molecular mechanisms in high anxiety. Based on the previously observed increase of OXPHOS protein expression in HAB mice [29], we used an antibody cocktail to assess simultaneously protein expression of all OXPHOS complexes in the hypothalamus, prefrontal cortex, and hippocampus. For the OXPHOS proteins that were quantified per brain region, we found no expression differences between HAB EH and NAB EH mice (Fig. 4a).

Increased oxidative stress has been linked with anxiety [49]. Furthermore, mitochondria-centered targeting of oxidative stress exerted anxiolytic effects in HAB mice [39]. Therefore, we examined the impact of the EH-driven anxiolytic effect in HAB mice on oxidative stress pathways. We analyzed protein markers of antioxidant pathways in all three regions of interest, including the antioxidant enzymes glutathione reductase (GSR), peroxiredoxin (PRX), catalase (CAT), and mitochondrial superoxide dismutase 2 (SOD2) and found no expression differences between HAB EH and HAB NH mice (Figs. 4b and S2). Additionally, to assess the overall oxidative status, we performed TAC assays in plasma (Fig. 4c) and brain (hypothalamus, prefrontal cortex, and hippocampus) (Fig. 4d) and observed no differences in total antioxidant capacity between HAB EH and HAB NH mice. Overall, these data indicate that EH has no impact on OXPHOS and oxidative stress in adult HAB male mice.

Due to the fact that the prefrontal cortex is particularly prone to oxidative damage [50], we went on to evaluate protein carbonylation, a marker of oxidative damage, in the prefrontal cortex and detected no changes in HAB EH vs. HAB NH mice (Fig. 4e). We found a negative correlation of carbonylation levels with the latency to the first entry and a trend towards a positive correlation with the number of entries in the light compartment in DaLi (Fig. 4e), suggesting that increased carbonylation correlates with lower anxiety-related behavior. This holds true only for HAB EH mice, as there is no significant correlation between HAB NH anxiety-related behavior and protein carbonylation (for entries Pearson *r* =  − 0.1235, *p* = 0.8157, and for latency to the light compartment Pearson *r* =  − 0.3753, *p* = 0.4634). Taken together, these results suggest that EH may mediate the association of oxidative damage and anxiety-related behavior in a high anxiety background in the prefrontal cortex.

### Early Handling (EH) Affects Glycolysis in the Hypothalamus

**Fig. 5:**
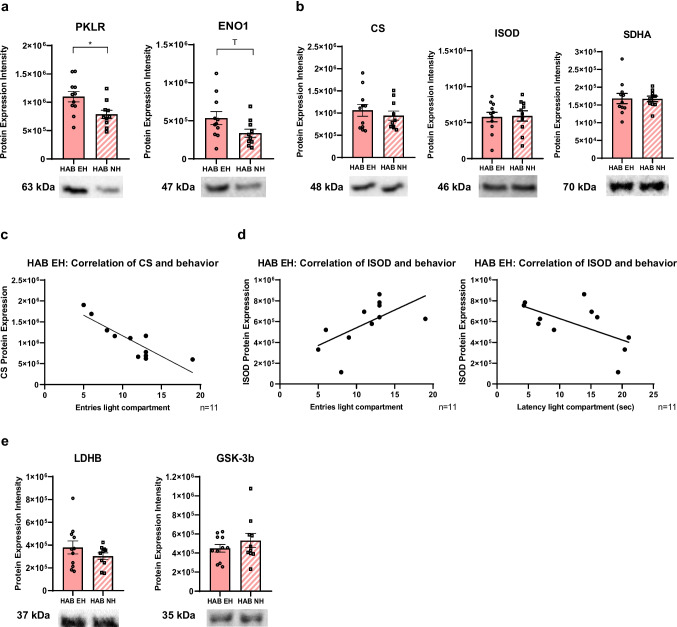
Early handling (EH) affects metabolism pathways in the hypothalamus. **a** Increased protein expression of PKLR (*p = 0.0150, Welch’s test) and a trend towards increased expression of ENO1 (*p* = 0.0661 (T), Welch’s test) in the hypothalamus of HAB EH (*n* = 11) vs. HAB NH (*n* = 10) mice. **b** No differences in protein expression of CS and ISOD and SDHA in the hypothalamus of HAB EH (*n* = 11) vs. HAB NH (*n* = 10) mice. **c** Hypothalamic CS expression strongly correlates with the number of entries in the DaLi in HAB EH mice (Pearson *r* =  − 0.8695, ***p = 0.0005). **d** Hypothalamic ISOD expression in HAB EH hypothalamus positively correlates with the number of entries (Pearson *r* = 0.6172, *p = 0.0431) and negatively correlates with the latency to the first entry in the light compartment (Pearson *r* =  − 0.6092, *p = 0.0467) in DaLi. **e** No differences in LDHB or in GSK-3b protein expression in the hypothalamus of HAB EH (*n* = 11) vs. HAB NH (*n* = 10) mice. Full Western blot data and loading control quantifications are provided in Figs. S3 and S4, respectively. HAB EH, early handling HAB; HAB NH, no handling HAB; T, trend

We then asked whether EH affects brain glycolysis by comparing the protein levels of the glycolysis enzymes glyceraldehyde 3-phosphate dehydrogenase (GAPDH), a-enolase (ENO1), and pyruvate kinase (PKLR) in the hypothalamus, prefrontal cortex, and hippocampus of HAB EH vs. HAB NH mice. We found increased glycolysis enzyme expression in the hypothalamus, namely increased PKLR expression and a trend towards increased ENO1 expression (Fig. 5a), while GAPDH levels were not significantly different in HAB EH compared to HAB NH mice.

To address whether the identified glycolysis changes impact on downstream metabolic pathways, we looked at the Krebs cycle, lactate production, and glycogen synthesis in the hypothalamus. We assessed the Krebs cycle by comparing the protein levels of Krebs cycle enzymes citric synthase (CS), isocitrate dehydrogenase (ISOD), and succinate dehydrogenase subunit A (SDHA) in HAB EH vs. HAB NH hypothalamus and found no expression differences between the two groups (Fig. 5b). Interestingly, there is a strong negative correlation of hypothalamic CS expression with the number of entries in the DaLi in HAB EH mice (Fig. 5c) but not in HAB NH mice (Pearson *r* =  − 0.4457, *p* = 0.1968). Furthermore, hypothalamic ISOD levels positively correlate with the number of entries and negatively correlate with the latency to the first entry in the light compartment in DaLi in HAB EH (Fig. 5d) but not in HAB NH mice (for entries Pearson *r* =  − 0.3861, *p* = 0.2704 and for latency Spearman *r* = 0.1636, *p* = 0.6567, respectively). These data suggest that EH induces a differential association with metabolism protein levels and anxiety-related behavior in HAB mice which is not present in NH mice. We also investigated lactate production by examining lactate dehydrogenase (LDHB), which catalyzes the conversion of the glycolysis endpoint pyruvate to lactate, and synthesis of glycogen, the storage form of glucose, by evaluating glycogen synthase kinase-3 beta (GSK-3b), which inhibits glycogen synthesis. We found no expression differences in the aforementioned proteins between the two groups (Fig. 5e). Overall, EH impacts the crosstalk of glycolysis and Krebs cycle in the hypothalamus.

### Early Handling (EH) Affects Mitochondrial Dynamics Protein Expression in the Hypothalamus

**Fig. 6:**
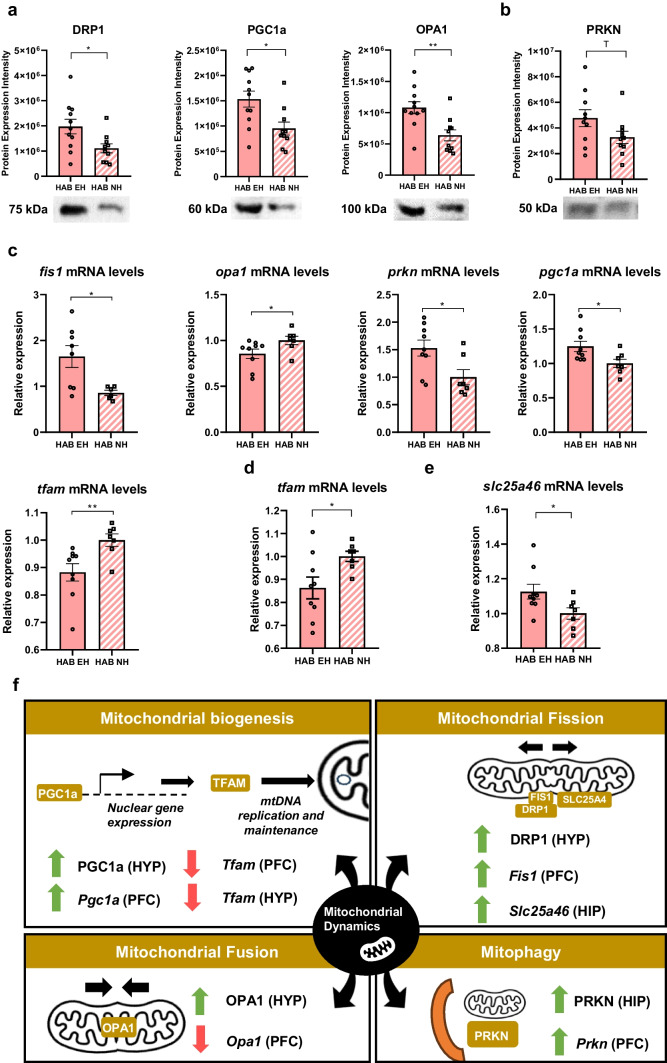
Early handling (EH) induces alterations in mitochondrial dynamics in adult HAB brain in a region-specific manner. **a** Increased protein expression of DRP1 (*p = 0.0221, Welch’s *t*-test), PGC1a (*p = 0.0102, Welch’s *t*-test) and OPA1 (**p = 0.0030, Welch’s *t*-test) in the hypothalamus of HAB EH (*n* = 11) vs. HAB NH (*n* = 10) male mice. **b** A trend towards increased protein expression of PRKN (*p* = 0.0832 (T), Welch’s *t*-test) in the hippocampus of HAB EH (*n* = 10) vs. HAB NH (*n* = 10) male mice. **c** Altered mRNA expression in the prefrontal cortex of HAB EH male mice. Increased *Fis1* (*p = 0.0121, Welch’s test), decreased *Opa1* (*p = 0.0246, Mann–Whitney test), increased *Prkn* (*p = 0.0289, Mann–Whitney test), increased *Pgc1a* (*p = 0.0224, Welch’s test), and decreased *Tfam* (**p = 0.0093, Welch’s test) mRNA levels in the prefrontal cortex of HAB EH vs. HAB NH male mice (HAB EH *n* = 9, HAB NH *n* = 7, for *Fis1* in hippocampus EH *n* = 8, HAB NH *n* = 6). **d** Decreased *Tfam* mRNA expression (*p = 0.0240, Welch’s test) in the hypothalamus of HAB EH (*n* = 9) vs. HAB NH (*n* = 7) male mice. **e** Increased *Slc25a46* mRNA expression (*p = 0.0362, Welch’s test) in the hippocampus of HAB EH (*n* = 9) vs. HAB NH (*n* = 7) male mice. **f** Schematic representation of different brain mitochondrial dynamics gene/protein regulatory networks affected by EH (green arrow: higher expression in HAB EH mice; red arrow: lower expression in HAB EH mice). Full Western blot data and loading control quantifications are provided in Figs. S3 and S4, respectively. HAB EH, early handling HAB; HAB NH, no handling HAB; HYP, hypothalamus; PFC, prefrontal cortex; HIP, hippocampus; T, trend

Recent work has implicated mitochondrial dynamics changes in anxiety [27, 28]. We, therefore, moved to explore whether EH-induced anxiolytic effects are mediated by mitochondrial dynamics alterations in the adult brain. To do so, we assessed protein expression changes in the mitochondrial dynamics machinery, including key players of mitochondrial fission (dynamin-1-like protein (DRP1), mitochondrial fission 1 protein (FIS1), solute carrier family 25 member 46 (SLC25A46), mitochondrial fission factor (MFF)), fusion (mitofusin 2 (MFN2), dynamin-like 120 kDa protein, mitochondrial (OPA1)), biogenesis (transcription factor A, mitochondrial (TFAM), peroxisome proliferator-activated receptor gamma coactivator 1-alpha (PGC1a)), and mitophagy (serine/threonine-protein kinase PINK1, mitochondrial (PINK1), E3 ubiquitin-protein ligase parkin (PRKN)) in the hypothalamus, prefrontal cortex, and hippocampus of HAB EH vs. HAB NH mice. We found that EH alters mitochondrial dynamics protein expression in the hypothalamus. In particular, EH increased the protein expression of the fission protein DRP1, the fusion protein OPA1 (long isoform detected in 100 kDa), and the mitochondrial biogenesis protein PGC1a in HAB male mice (Fig. 6a). No differences were found in the short OPA1 isoform (detected in 80 kDa). No mitochondrial dynamics protein expression changes were observed in the prefrontal cortex and hippocampus with the exception of a trend towards increased expression of the mitophagy protein PRKN in the hippocampus (Fig. 6b). These data indicate a hypothalamus-centered effect of EH in mitochondrial dynamics protein regulation in adulthood.

### Early Handling (EH) Affects Mitochondrial Dynamics Gene Expression Predominantly in the Prefrontal Cortex

Having observed protein expression differences in mitochondrial dynamics after EH, we looked further into gene expression alterations in the mitochondrial dynamics machinery by comparing the mRNA levels of the same mitochondrial dynamics players which were assessed at the protein level in the hypothalamus, prefrontal cortex, and hippocampus of HAB EH vs. HAB NH mice.

In the prefrontal cortex, we found increased mRNA levels of the fission gene *Fis1* and decreased mRNA levels of the fusion gene *Opa1* in HAB EH mice. EH also increased mRNA expression of the mitophagy gene *Prkn* in the HAB prefrontal cortex. Furthermore, the mitochondrial biogenesis mediators *Pgc1a* and *Tfam* mRNA levels were increased and decreased, respectively, in HAB EH mice (Fig. 6c). *Tfam* mRNA levels were also decreased in the hypothalamus of HAB EH mice (Fig. 6d). The observed fission changes in the prefrontal cortex were accompanied by increased mRNA levels of the fission mediator *Slc25a46* in the hippocampus (Fig. 6e). Overall, EH results in altered mitochondrial dynamics gene expression signatures predominantly in the prefrontal cortex.

## Discussion

In this work, we aimed to elucidate the effects of EH in high anxiety mice at the behavioral and the molecular levels. At the behavioral level, we report that EH exerts anxiolytic effects in adult HAB male mice, as demonstrated by the increased number of entries and decreased latency to the first entry in the DaLi light compartment (Fig. 1b). Notably, the EH-induced anxiolytic effects in DaLi are specific for HAB mice, as no EH-driven behavioral changes are observed in NAB mice. This suggests that the beneficial effects of EH are more pronounced in vulnerable mice deriving from a high anxiety background and highlights that early life interventions are crucial for shaping adult life behaviors particularly in at-risk populations, which warrants further investigation for translational implementation. Furthermore, we found that EH decreases SPA index in HAB EH compared to HAB NH male mice on PND 31. However, the SPA index exceeds 50% in both ΗΑΒ groups as well as in NAB mice, which showcases an overall lack of social avoidance in both HAB and NAB mouse lines (Fig. S1a).

As maternal behavior has been reported to affect behavioral outcomes in adult offspring [51] and the EH paradigm transiently interferes with the dam-pup bond, we asked whether dam behavior is affected by EH in HAB and NAB mice. We observed no significant changes in a range of maternal behaviors scored during EH (Fig. 2b). This holds true both for HAB and NAB dams and may indicate that the behavioral and molecular pup outcomes after EH are due to the separation of the pups from their dam and not due to a change in dam behavior during EH. Using the same EH protocol, Luchetti et al. reported differences in maternal nursing upon EH in NMRI dams [8], whereas Akatsu et al. observed increased pup licking in C57BL/6 dams [9]. However, these data were statistically analyzed including additional intervention groups (cross-fostering and prenatal stress, respectively). In a similar study conducted in Wistar rats, EH induced increased licking behavior in dams [52]. The different organisms/mouse strains used may account for the divergent maternal behavior results across studies.

When we analyzed maternal behavior in basal (not handled) HAB and NAB dams, we found that HAB dams exhibit increased maternal care compared to NAB dams (Fig. 3a, b). Previous studies in HAB/LAB mice have indicated that HAB dams show increased arched-back nursing and thus maternal care along with higher mRNA vasopressin levels in hypothalamic paraventricular nucleus compared to LAB dams. However, cross-fostering between HAB and LAB lines only moderately affects the inherent anxiety phenotype of the offspring [53]. In a cross-breeding study of offspring deriving from HAB female and LAB male mice, pups that grew up with a foster LAB mother showed decreased anxiety-related behavior compared to controls, indicating that line-specific maternal care characteristics do affect adult future outcomes in this mouse model [54]. As the maternal behavior data were based on a relatively low number of available dams (*n* = 4–6 per group), these results need to be substantiated in larger HAB and NAB dam cohorts.

At the molecular level, we dissected the molecular underpinnings of the EH-driven anxiolytic effects of pups in adulthood, by performing the same analyses in three brain regions. The prefrontal cortex and the hippocampus were selected as they constitute core brain areas involved in emotional processing, [55] and our previous work has shown altered metabolism, mitochondrial pathways, and oxidative stress both in the cortex [29, 30] and hippocampus [32] of HAB mice. We also included the hypothalamus which modulates fear conditioning and extinction that is crucial for anxiety-related behavior [33]. Our results show that ΕH does not alter OXPHOS protein subunit expression in all three brain regions studied (Fig. 4a). Moreover, the oxidative status of HAB mice after EH remains unchanged, as evidenced by the lack of changes in oxidative stress markers and TAC in all three brain regions assessed (Figs. 4b, S2, and 4d), in plasma TAC (Fig. 4c) and in protein carbonylation in the prefrontal cortex (Fig. 4e) in HAB EH compared to HAB NH mice. Intriguingly, protein carbonylation levels negatively correlate with anxiety-related behavior (Fig. 4f) in HAB EH but not in HAB NH mice. In HAB EH mice, the more oxidative damage in the form of protein carbonylation, the less time HAB mice need to enter for the first time the Dali light compartment and they more often do so. This observation along with the lack of correlation between anxiety-related behavior and protein carbonylation in HAB NH mice and the corroborated findings of increased oxidative stress in HAB mice [29, 30] may suggest that EH exerts a prophylactic effect in early life from the cumulative oxidative stress contributing to increased anxiety-related behavior in adulthood. Given that stressful events throughout the lifetime promote oxidative stress imbalance [56], we hypothesize that EH confers resilience to increased oxidative stress in high anxiety populations in later life which should be further studied.

Besides OXPHOS, we then moved to investigate cytoplasmic metabolism by assessing glycolysis enzyme levels across all three brain regions. We report an increased protein expression of glycolytic enzymes exclusively in the hypothalamus in HAB EH mice (Fig. 5b) which led us to assess the fate of pyruvate in downstream pathways, including the Krebs cycle, lactate production, and glycogen synthesis in this region. Although no expression differences were observed in Krebs cycle enzymes in HAB EH vs. HAB NH mice, hypothalamic CS (Fig. 5c) and ISOD (Fig. 5d) levels negatively and positively correlate with anxiety-related behavior, respectively, in HAB EH but not in HAB NH mice. This may suggest that EH selectively affects the crosstalk of glycolysis and Krebs cycle and contributes to the sensitivity of metabolic regulation of behavioral outcomes in high anxiety.

The most prominent molecular changes after EH were observed in mitochondrial dynamics processes in the hypothalamus of HAB EH mice, where increased protein expression of the fission protein DRP1, the fusion protein OPA1, and the mitochondrial biogenesis protein PGC1a in HAB mice were identified (Fig. 6a). DRP1 is a GTPase which when recruited to mitochondria executes fission by self-polymerization around the mitochondrial membrane followed by mitochondrial constriction [57]. Independently of mitochondrial fission, DRP1 has been also shown to mediate post-synaptic endocytosis and formation of dendrites in mice [58] and to be required synergistically with PRKN for maintaining mitochondrial integrity [59]. ΟPA1 is another GTPase that drives the fusion of inner mitochondrial membranes [60] which is expressed in higher levels in HAB compared to LAB cortical synaptosomes [29]. In order to elicit the inner mitochondrial membrane fusion, OPA1 is cleaved, degrading long OPA1 isoforms into short OPA1 isoforms [26]. The increase in OPA1 levels observed here refers to the long OPA1 isoform. PGC1a is a transcriptional coactivator promoting mitochondrial biogenesis [61] with additional roles in energy metabolism modulation [62].

The observed glycolysis and mitochondrial dynamics protein changes upon EH in adulthood were mainly hypothalamus-specific. The hypothalamus is an epicenter of food intake control and, accordingly, of energy homeostasis regulation [63]. Therefore, early life interventions may have a more profound effect and functional relevance in energy-regulating pathways such as glycolysis and mitochondria quality control in the hypothalamus compared to other brain regions [64]. Also, as a crucial component of the hypothalamus–pituitary–adrenal axis, the hypothalamus may respond to early life interventions by modulating adult life outcomes [65].

At the gene expression level, the most prominent EH-induced changes were found in the prefrontal cortex, a brain region which has been shown to be prone to emotionally regulated mitochondrial transcription control [66]. We report increased mRNA levels of the fission player *Fis1* accompanied by decreased mRNA levels of the fusion player *Opa1* in HAB EH compared to HAB NH mice, suggesting a shift from fusion to fission upon EH in adulthood. We also found increased *Prkn* levels in HAB EH vs. HAB NH mice, pointing towards increased mitophagy upon EH. Moreover, we observed mRNA levels of *Pgc1a* which promotes mitochondrial biogenesis along with decreased *Tfam* mRNA levels in HAB EH vs. HAB NH mice, indicating alterations in mitochondrial transcription control (Fig. 6c). Interestingly, mRNA *Tfam* levels were also increased in the hypothalamus of HAB EH vs. HAB NH mice (Fig. 6d), suggesting a widespread induction of transcriptional regulation upon EH in the adult HAB brain. Addressing collectively the observed changes, we hypothesize that EH fosters a shift towards fission and mitophagy in the adult HAB prefrontal cortex. This, along with the ΕH-induced changes in the transcriptional regulators *Pgc1a* and *Tfam*, may indicate an overall remodeling of mitochondria quality control in order to address cumulative mitochondrial damage or dysfunctional mitochondria by either excising these mitochondrial populations through fission and/or removing them by mitophagy. Therefore, EH may enhance the robustness of mitochondrial quality control in adulthood, and consequently, a pool of functional mitochondria being able to optimally perform energy-dependent processes in the prefrontal cortex may contribute to the observed EH-induced anxiolytic effects in HAB mice.

Intriguingly, OPA1 protein levels were increased in the hypothalamus (Fig. 6a), while *Opa1* mRNA levels were decreased in the prefrontal cortex (Fig. 6c) of HAB EH vs. HAB NH mice. This is not surprising as the regulation of mitochondrial dynamics mechanisms may be region-specific with each brain region having a unique mitochondrial gene/protein signature according to its specific bioenergetic needs. For instance, differential mitochondrial gene expression signatures have been observed in the rodent prefrontal cortex compared to other brain regions upon exposure to chronic stress [66]. At the molecular level, mRNA levels do not correlate with protein levels of a certain gene/protein due to the complex nature of mammalian transcriptional control [67]. Overall, the diverse, EH-induced, brain region-specific mRNA and protein mitochondrial dynamics profiles reported here can be attributed to the multifactorial regulation of the brain translational machinery, the circuits and connectivity between different brain regions related to emotional regulation, and the distinct bioenergetic profiles per region.

Although mitochondrial alterations in brain and peripheral material have been reported when investigating the impact of early life adversity in rodents and humans [68–70], our knowledge of how mitochondria mediate the effects of beneficial early life interventions in adulthood is very limited. We have previously demonstrated that pharmacologically targeting mitochondria in high anxiety exerts anxiolytic effects, as HAB mice chronically treated with the mitochondrial-targeted antioxidant MitoQ show decreased anxiety-related behavior in DaLi compared to untreated HAB counterparts. This anxiolytic effect was specific for HAB mice provided that no anxiolytic effects were observed in mouse strains not bred for high anxiety [39].

Importantly, the aforementioned studies mainly focus on mitochondrial energy metabolism and oxidative stress pathways whereas mitochondrial dynamics alterations upon beneficial early life interventions have not been elucidated. The innovation of the current work lies on the fact that we focus οn how mitochondrial dynamics is affected after an early life intervention in a background of high trait anxiety. To the best of our knowledge, this is the first study to assess the effects of EH in a high anxiety background, investigate mitochondrial readouts, and reveal mitochondrial dynamics changes in adulthood upon EH. We hypothesize that the increased expression of fission, fusion, and biogenesis molecular machinery components reported here may indicate an activation of mitochondrial quality control in early life by EH, which ensures an optimal function of the brain mitochondrial network to fuel local energy-dependent processes and prevents mitochondrial damage in adulthood.

Taken together, we report that EH is anxiolytic in a high anxiety background and that the EH-induced anxiolytic effects are accompanied by brain region-specific changes in energy metabolism and mitochondrial dynamics. Additional studies are required to substantiate a causal role of mitochondria and mitochondrial dynamics in modulating the effects of EH, such as gene expression interference and/or rescue experiments. Furthermore, assessing brain mitochondrial morphology and shape along with the proximity of mitochondria to synapses using microscopy approaches as well as investigating the observed mitochondrial dynamics changes in a cell-type dependent manner will shed light on mitochondrial quality control mechanisms and heterogeneity in different brain cell types [71]. The molecular analysis of this current work has focused on male mice. As there is an overrepresentation of male subjects in animal studies [72] and sex differences do exist when assessing anxiety-related behavior in rodents and humans [73–75], we will next focus on examining EH effects in high anxiety female mice to address sex-specific, EH-induced effects. Future directions also include investigating the potential of pharmacologically manipulating the mitochondrial dynamics machinery [76] to achieve anxiolytic effects in a high anxiety background in vivo.

## Supplementary Information

Below is the link to the electronic supplementary material.

Supplementary file1: Behavioral outcomes after early handling (EH) in HAB and NAB male mice a. SPAT behavioral scores for total interaction time and SPA index (HAB EH n=15, HAB NH n=15, NAB EH n=15 and NAB NH n=5). EH significantly decreases SPA index HAB EH vs. HAB NH mice (*p=0.0110). b. EH does not affect time spent in the DaLi light compartment in HAB and NAB mice (HAB EH n=20, HAB NH n=17, NAB EH n=14 and NAB NH n=7). c. EH-induced anxiolytic effects in DaLi number of entries and latency to the first entry to the light compartment are not driven by specific litters as shown by plotting these two parameters for HAB EH and HAB NH mice. Each symbol per group denotes mice from a different litter. d. EH does not affect OFT behavioral readouts in HAB and NAB mice, including time spent in the center, number of entries in the center, latency to the first entry in the center and line crossings (HAB EH n=20, HAB NH n=17, NAB EH n=15 and NAB NH n=7). e. FST behavioral scores for time floating, latency to the first floating event, struggling and swimming (HAB EH n=20, HAB NH n=17, NAB EH n=15 and NAB NH n=7). There is a trend towards decreased latency to the first floating event in HAB EH vs. HAB NH male mice (p=0.0566 (T)). HAB EH: early handling HAB, HAB NH: no handling HAB, NAB EH: early handing NAB, NAB NH: no handling NAB, PND: post-natal day T: trend (PDF 282 KB)

Supplementary file2: Quantification of oxidative stress protein markers in HAB EH vs. HAB NH male mice No protein expression differences were found in a. hypothalamus b. prefrontal cortex and c. hippocampus (HAB EH n=11, HAB NH n=10, for PRX in hippocampus EH n=9, HAB NH n=10). HAB EH: early handling HAB, HAB NH: no handling HAB (PDF 176 KB)

Supplementary file3: Full Western blot data of proteins assessed in HAB EH vs. HAB NH mice in a. hypothalamus b. prefrontal cortex and c. hippocampus EH: early handling, NH: no handling (PDF 237 KB)

Supplementary file4: Quantification of Ponceau membrane staining for Western blot data of proteins assessed in HAB EH vs. HAB NH mice in a. hypothalamus b. prefrontal cortex and c. hippocampus HAB EH: early handling HAB, HAB NH: no handling HAB, HYP: hypothalamus, PFC: prefrontal cortex, HIP: hippocampus, CI: OXPHOS complex I, CIII: OXPHOS complex III (PDF 784 KB)

Supplementary file5: Maternal behaviors observed during the EH protocol (DOCX 17.7 KB)

Supplementary file6: Primary antibodies and corresponding dilutions along with secondary antibodies used in Western blots (PDF 164 KB)

Supplementary file7: Primers used in qRT-PCRs. Gene and full gene names, sequences of the forward (F’) and reverse (R’) primers, length of the product acquired, melting temperature (Tm) and GC% content (PDF 172 KB)

## Data Availability

All data required for interpretation of the results are provided in the manuscript and in the Supplementary Material. Additional raw data are available from the authors upon request.

## Abbreviations

DaLi: Dark-light box
EH: Early handling
EPM: Elevated plus maze
FST: Forced swim test
HAB: High anxiety-related behavior
LAB: Low anxiety-related behavior
NAB: Normal anxiety-related behavior
NH: No handling
OFT: Open field test
OXPHOS: Oxidative phosphorylation
PND: Post-natal day
SPA: Social preference-avoidance
SPAT: Social preference-avoidance test
