# Supplementary figures and images for: Early Handling Exerts Anxiolytic Effects and Alters Brain Mitochondrial Dynamics in Adult High Anxiety Mice
Source: Mol Neurobiol. 2024 May 18;61(12):10593–612. doi: 10.1007/s12035-024-04116-5 (PMC11584496; doi:10.1007/s12035-024-04116-5)

**Figure S1**

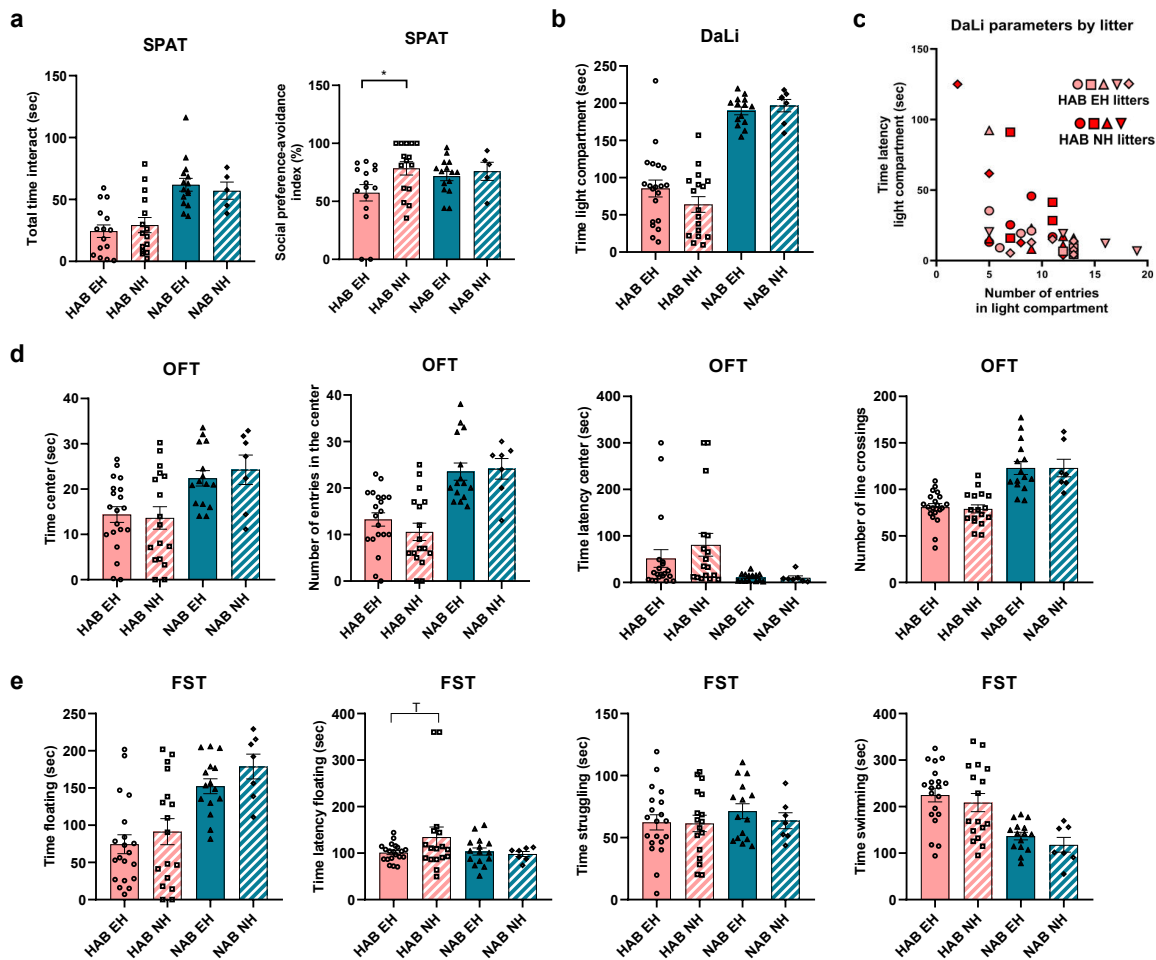

Supplement: Supplementary file 1 — Supplementary file1: Behavioral outcomes after early handling (EH) in HAB and NAB male mice a. SPAT behavioral scores for total interaction time and SPA index (HAB EH n=15, HAB NH n=15, NAB EH n=15 and NAB NH n=5). EH significantly decreases SPA index HAB EH vs. HAB NH mice (*p=0.0110). b. EH does not affect time spent in the DaLi light compartment in HAB and NAB mice (HAB EH n=20, HAB NH n=17, NAB EH n=14 and NAB NH n=7). c. EH-induced anxiolytic effects in DaLi number of entries and latency to the first entry to the light compartment are not driven by specific litters as shown by plotting these two parameters for HAB EH and HAB NH mice. Each symbol per group denotes mice from a different litter. d. EH does not affect OFT behavioral readouts in HAB and NAB mice, including time spent in the center, number of entries in the center, latency to the first entry in the center and line crossings (HAB EH n=20, HAB NH n=17, NAB EH n=15 and NAB NH n=7). e. FST behavioral scores for time floating, latency to the first floating event, struggling and swimming (HAB EH n=20, HAB NH n=17, NAB EH n=15 and NAB NH n=7). There is a trend towards decreased latency to the first floating event in HAB EH vs. HAB NH male mice (p=0.0566 (T)). HAB EH: early handling HAB, HAB NH: no handling HAB, NAB EH: early handing NAB, NAB NH: no handling NAB, PND: post-natal day T: trend (PDF 282 KB) [file 12035_2024_4116_MOESM1_ESM.pdf]

Figure S2

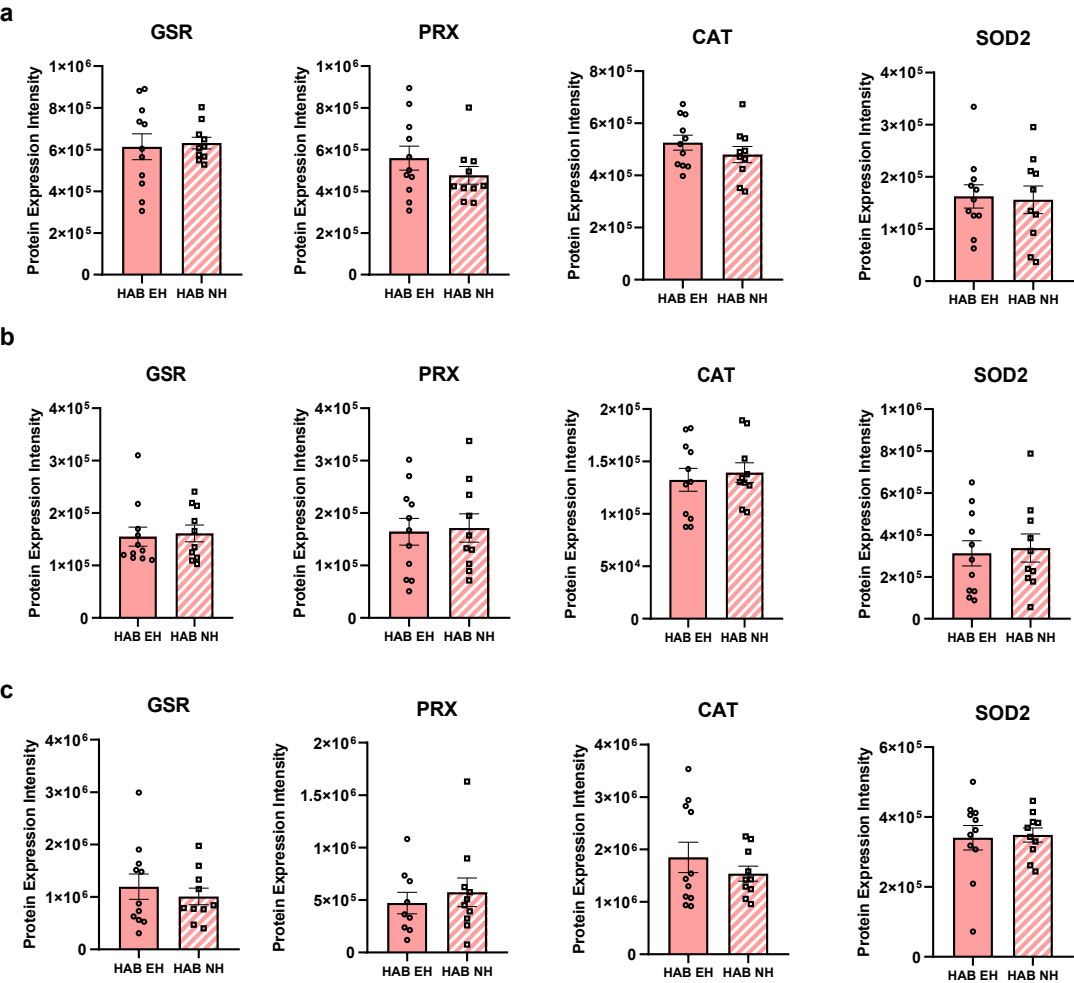

Supplement: Supplementary file 2 — Supplementary file2: Quantification of oxidative stress protein markers in HAB EH vs. HAB NH male mice No protein expression differences were found in a. hypothalamus b. prefrontal cortex and c. hippocampus (HAB EH n=11, HAB NH n=10, for PRX in hippocampus EH n=9, HAB NH n=10). HAB EH: early handling HAB, HAB NH: no handling HAB (PDF 176 KB) [file 12035_2024_4116_MOESM2_ESM.pdf]

**Figure S3**

**a. Hypothalamus**

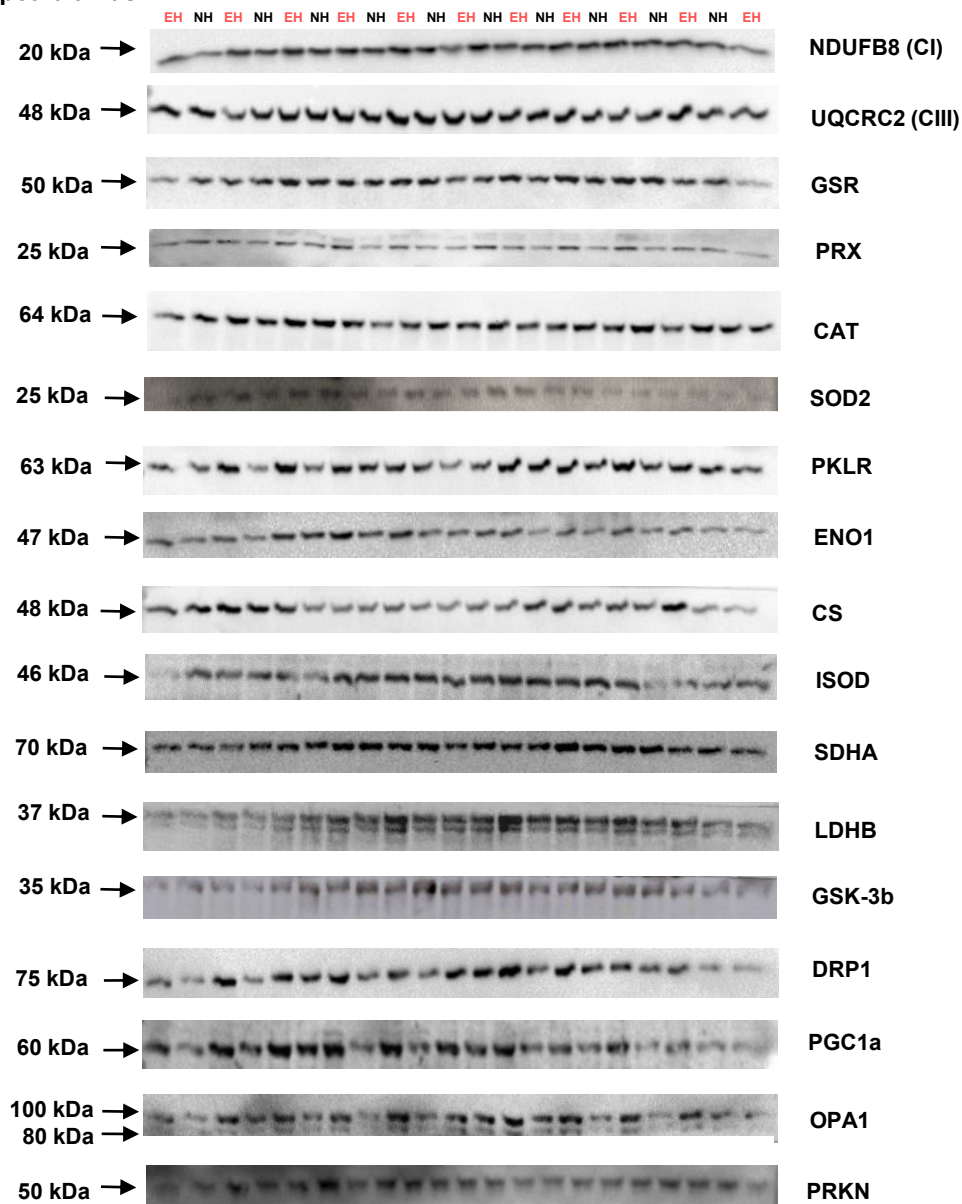

**b. Prefrontal cortex**

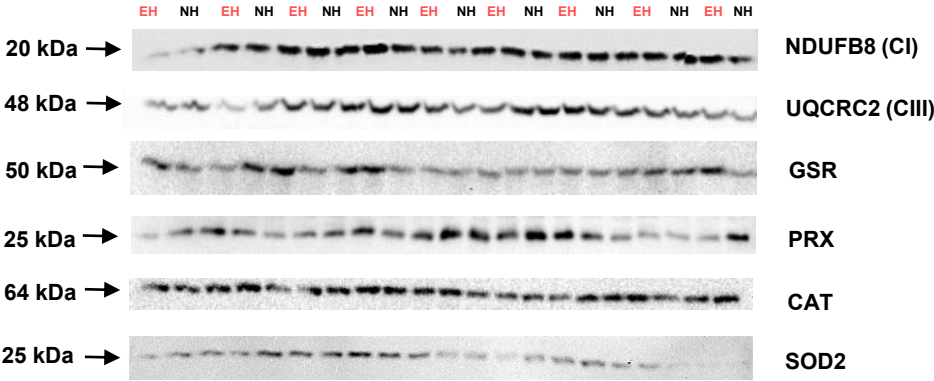

c. Hippocampus

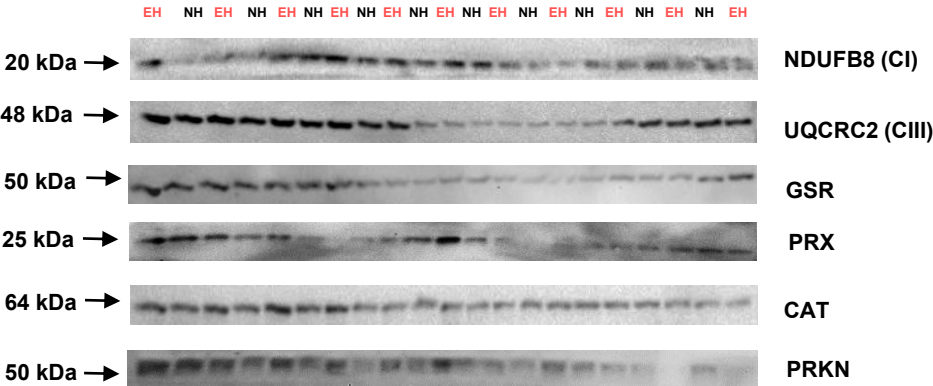

Supplement: Supplementary file 3 — Supplementary file3: Full Western blot data of proteins assessed in HAB EH vs. HAB NH mice in a. hypothalamus b. prefrontal cortex and c. hippocampus EH: early handling, NH: no handling (PDF 237 KB) [file 12035_2024_4116_MOESM3_ESM.pdf]
