# Supplementary material for: Early Handling Exerts Anxiolytic Effects and Alters Brain Mitochondrial Dynamics in Adult High Anxiety Mice
Source: Mol Neurobiol. 2024 May 18;61(12):10593–612. doi: 10.1007/s12035-024-04116-5 (PMC11584496; doi:10.1007/s12035-024-04116-5)

Figure S4

a. Hypothalamus

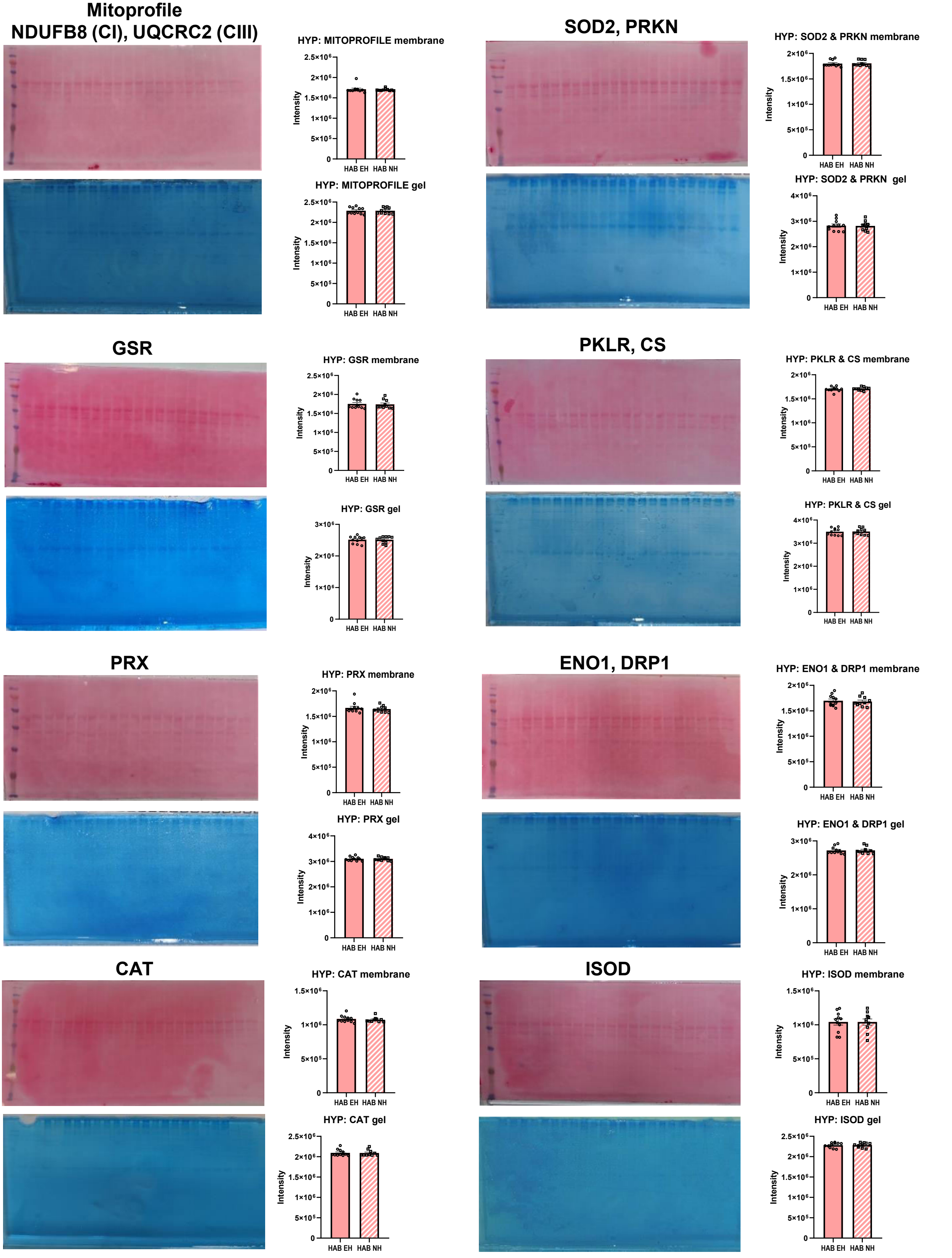

SDHA

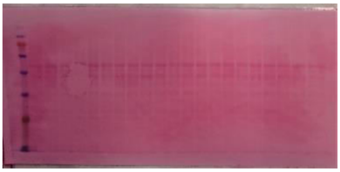

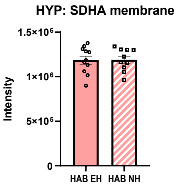

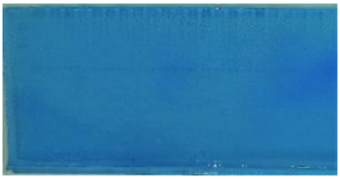

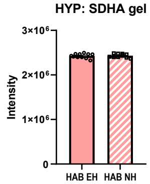

GSK-3b

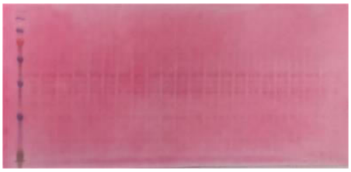

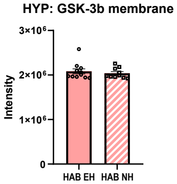

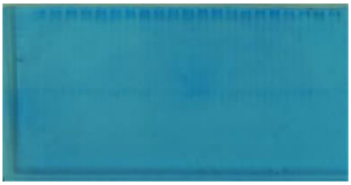

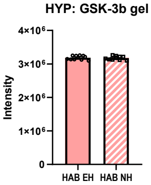

LDHB, OPA1, PGC1a

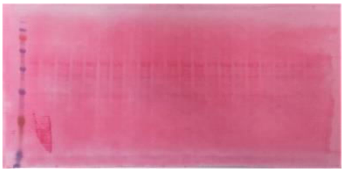

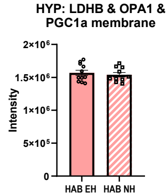

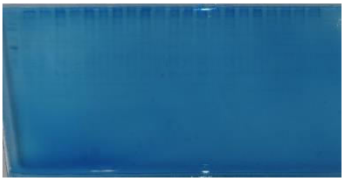

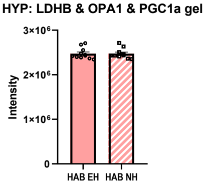

b. Prefrontal cortex

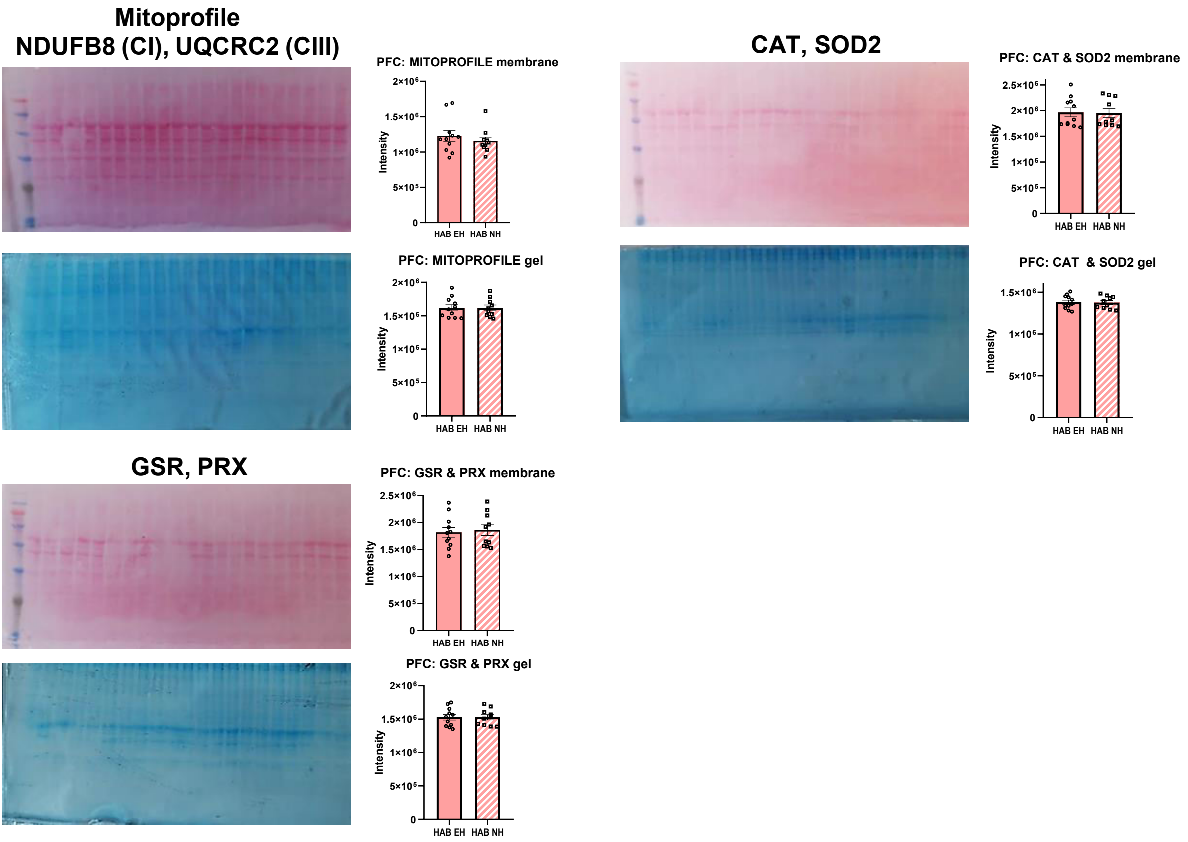

c. Hippocampus

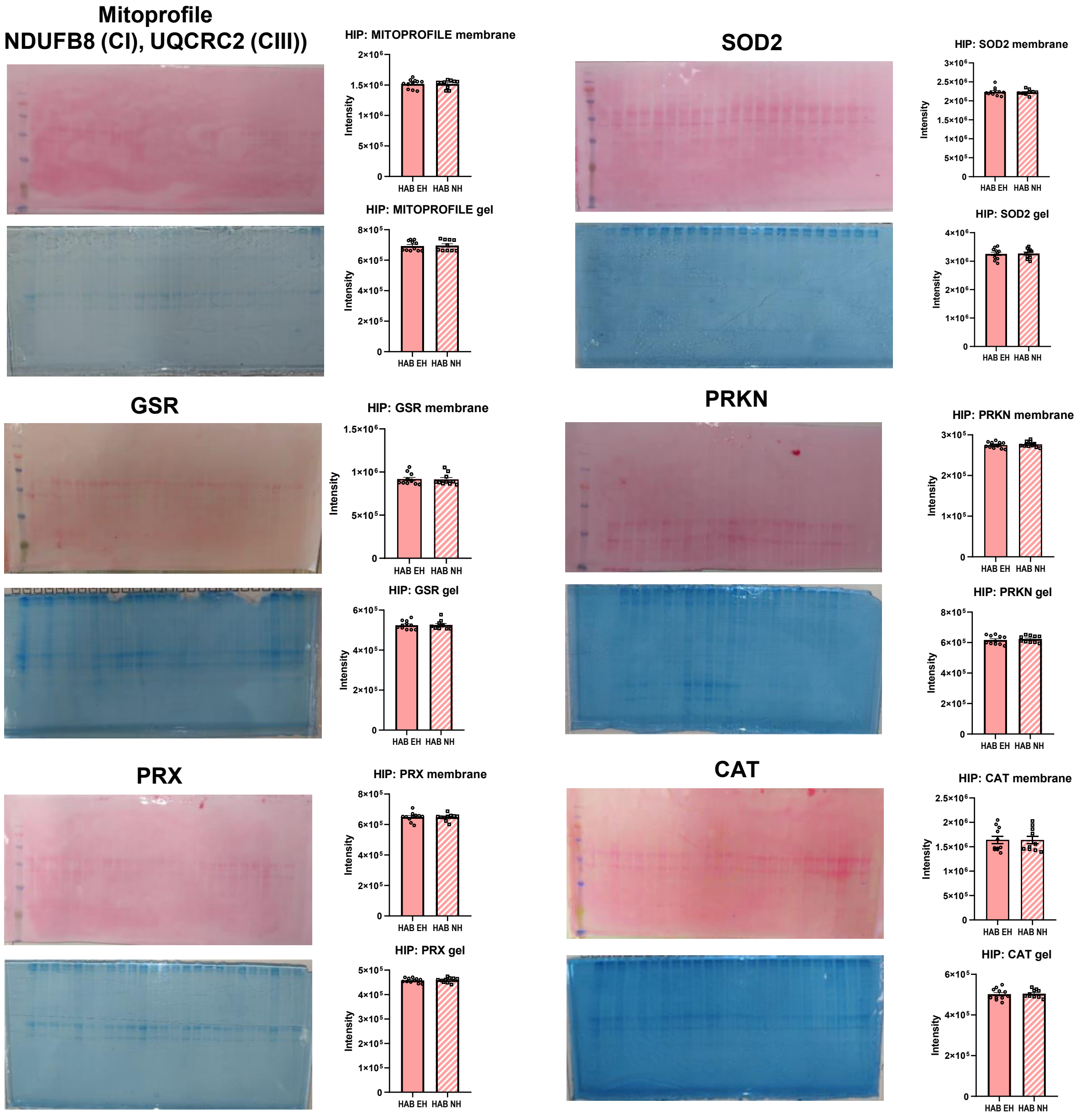

Supplement: Supplementary file 4 — Supplementary file4: Quantification of Ponceau membrane staining for Western blot data of proteins assessed in HAB EH vs. HAB NH mice in a. hypothalamus b. prefrontal cortex and c. hippocampus HAB EH: early handling HAB, HAB NH: no handling HAB, HYP: hypothalamus, PFC: prefrontal cortex, HIP: hippocampus, CI: OXPHOS complex I, CIII: OXPHOS complex III (PDF 784 KB) [file 12035_2024_4116_MOESM4_ESM.pdf]
