# Supplementary material for: Early Handling Exerts Anxiolytic Effects and Alters Brain Mitochondrial Dynamics in Adult High Anxiety Mice
Source: Mol Neurobiol. 2024 May 18;61(12):10593–612. doi: 10.1007/s12035-024-04116-5 (PMC11584496; doi:10.1007/s12035-024-04116-5)
Supplement: Supplementary file 5 — Supplementary file5: Maternal behaviors observed during the EH protocol (DOCX 17.7 KB) [file 12035_2024_4116_MOESM5_ESM.docx]

| **Specific behavior** | **Description of behavior** | **Category of behavior** |
| --- | --- | --- |
| **Licking-grooming (LG)** | Dam touches the pup's body with her tongue or handles a pup's body with her forepaws or nose | Active caring behavior |
| **Arched-back nursing** **(ABN)** | Dam presents an upright dorsal arch posture with a depressed head over the pups |  |
| **Nursing** **(NU)** | Dam presents either a prone or upright sitting or sideways lying while being immobile over the pups | Passive caring behavior |
| **Nest building** **(NE)** | Dam collects and/or handles nesting material with mouth or forepaws while in contact with pups or without |  |
| **Self-maintenance** **(SM)** | Dam licks, brushes or scratches her fur or paws with tongue or paws, eats and drinks water | Self-maintenance |
| **Exploration** **(EXPL)** | Dam climbs with all four paws attached to the cage lid or burrows in cage out of nest or explores the cage out of the nest | Neglecting behavior |
| **Inactivity (IN)** | Dam sleeps or lies still out of the nest |  |

Adapted from Chourbaji et al. 2011

Chourbaji S, Hoyer C, Richter SH, Brandwein C, Pfeiffer N, Vogt MA, Vollmayr B, Gass P (2021) Differences in mouse maternal care behavior - is there a genetic impact of the glucocorticoid receptor? PLoS One 6:e19218. <https://doi.org/10.1371/journal.pone.0019218>
