# Supplementary material for: Early Handling Exerts Anxiolytic Effects and Alters Brain Mitochondrial Dynamics in Adult High Anxiety Mice
Source: Mol Neurobiol. 2024 May 18;61(12):10593–612. doi: 10.1007/s12035-024-04116-5 (PMC11584496; doi:10.1007/s12035-024-04116-5)
Supplement: Supplementary file 6 — Supplementary file6: Primary antibodies and corresponding dilutions along with secondary antibodies used in Western blots (PDF 164 KB) [file 12035_2024_4116_MOESM6_ESM.pdf]

Table S2

Function	Primary antibody	Full protein name	Manufacturer	Dilution	Secondary antibody
**OXPHOS**	**Mitoprofile**	**CI complex: NDUFB8<br>CII complex: SDHB<br>CIII complex: UQCRC2<br>CIV complex: MTCO1<br>CV complex: ATP5A**	MS604, Abcam	1:800	Anti-mouse (#170-6516, Bio-Rad)
**Antioxidant defense**	**GSR**	**Glutathione Reductase**	sc-133245, Santa Cruz Biotechnology	1:500	Anti-mouse (#170-6516, Bio-Rad)
	**PRX**	**Peroxiredoxin**	sc-137222, Santa Cruz Biotechnology	1:200	Anti-mouse (#170-6516, Bio-Rad)
	**CAT**	**Catalase**	sc-271803, Santa Cruz Biotechnology	1:500	Anti-mouse (#170-6516, Bio-Rad)
	**SOD2**	**Superoxide Dismutase 2**	sc-137254, Santa Cruz Biotechnology	1:200	Anti-mouse (#170-6516, Bio-Rad)
**Glycolysis**	**ENO1**	**Alpha-enolase**	sc-100812, Santa Cruz Biotechnology	1:200	Anti-mouse (#170-6516, Bio-Rad)
	**GAPDH**	**Glyceraldehyde-3-Phosphate Dehydrogenase**	sc-32233, Santa Cruz Biotechnology	1:500	Anti-mouse (#170-6516, Bio-Rad)
	**PKLR**	**Pyruvate Kinase, Liver**	sc-166228, Santa Cruz Biotechnology	1:800	Anti-mouse (#170-6516, Bio-Rad)
**Lactate metabolism**	**LDHB**	**Lactate Dehydrogenase B**	NB110-57160, Novusbio	1:2000	Anti-rabbit (20402-1, BIOTIUM)
**Glycogen metabolism**	**GSK-3b**	**Glycogen synthase kinase-3 beta**	9315, Cell Signaling Technology	1:500	Anti-rabbit (20402-1, BIOTIUM)
**Krebs cycle**	**CS**	**Citrate synthase, mitochondrial**	GTX110624, GeneTex	1:1000	Anti-rabbit (20402-1, BIOTIUM)
	**ISOD**	**Isocitrate dehydrogenase**	ab113232, Abcam	1:500	Anti-rabbit (20402-1, BIOTIUM)

Table S2

	**SDHA**	**Succinate dehydrogenase**	sc-98253, Santa Cruz Biotechnology	1:500	Anti-rabbit (20402-1, BIOTIUM)
**Fission**	**DRP1**	**Dynamin-1-like protein**	sc-271583, Santa Cruz Biotechnology	1:500	Anti-mouse (#170-6516, Bio-Rad)
	**FIS1**	**Mitochondrial fission 1 protein**	10956-1-AP, Proteintech	1:1000	Anti-mouse (#170-6516, Bio-Rad)
	**SLC25A46**	**Mitochondrial outer membrane solute carrier protein**	12277-1-AP, Proteintech	1:500	Anti-rabbit (20402-1, BIOTIUM)
	**MFF**	**Mitochondrial Fission Factor**	17090-1-AP, Proteintech	1:10000	Anti-rabbit (20402-1, BIOTIUM)
**Fusion**	**MFN2**	**Mitofusin 2**	12186-1-AP, Proteintech	1:2000	Anti-rabbit (20402-1, BIOTIUM)
	**OPA1**	**Dynamin-like 120 kDa protein, mitochondrial**	66583-1-Ig, Proteintech	1:500	Anti-mouse (#170-6516, Bio-Rad)
**Mitophagy**	**PRKN**	**E3 ubiquitin-protein ligase parkin**	sc-32282, Santa Cruz Biotechnology	1:500	Anti-mouse (#170-6516, Bio-Rad)
	**PINK1**	**Serine/threonine-protein kinase PINK1**	23274-1-AP, Proteintech	1:500	Anti-rabbit (20402-1, BIOTIUM)
**Biogenesis**	**TFAM**	**Transcription Factor A, mitochondrial**	ab131607, Abcam	1:1500	Anti-rabbit (20402-1, BIOTIUM)
	**PGC1a**	**Peroxisome Proliferator-Activated Receptor Gamma Coactivator-1 alpha**	66369-1-Ig, Proteintech	1:2000	Anti-mouse (#170-6516, Bio-Rad)
