# Supplementary material for: Early Handling Exerts Anxiolytic Effects and Alters Brain Mitochondrial Dynamics in Adult High Anxiety Mice
Source: Mol Neurobiol. 2024 May 18;61(12):10593–612. doi: 10.1007/s12035-024-04116-5 (PMC11584496; doi:10.1007/s12035-024-04116-5)
Supplement: Supplementary file 7 — Supplementary file7: Primers used in qRT-PCRs. Gene and full gene names, sequences of the forward (F’) and reverse (R’) primers, length of the product acquired, melting temperature (Tm) and GC% content (PDF 172 KB) [file 12035_2024_4116_MOESM7_ESM.pdf]

Table S3

Function	Primer	Full gene name	Sequence (5'->3')		Tm	GC%	Amplicon size (bp)
**Reference gene**	***Srp14***	**Signal Recognition Particle 14**	F'	CAGCGTGTTCATCACCCTCAA	60.88	52.38	109
			R'	GGCTCTCAACAGACACTTGTTTT	59.62	43.48	
**Fission**	***Mff***	**Mitochondrial Fission Factor**	F'	TCGGGTCTGTCCTCCCCATA	61.59	60	145
			R'	CAACACAGGTCTGCGGTTTTCA	61.83	50	
	Fis1	**Mitochondrial Fission 1 protein**	F'	CAAAGAGGAACAGCGGGACT	59.96	55	95
			R'	ACAGCCCTCGCACATACTTT	59.68	50	
	Slc25a46	**Mitochondrial outer membrane solute carrier protein**	F'	TCTGACGTTATACTTTACCC	51.78	40	137
			R'	CAGTCTCTCATTCCCTCATA	53.25	45	
	Drp1	**Dynamin-related protein 1**	F'	TGACCAAAGTACCTGTAGGCG	59.73	52.38	229
			R'	GCATCAGTACCCGCATCCAT	60.25	55	
**Fusion**	***Opa1***	**Dynamin-like 120 kDa protein**	F'	ACCTTGCCAGTTTAGCTCCC	59.96	55	82
			R'	TTGGGACCTGCAGTGAAGAA	59.16	50	
	Mfn2	**Mitofusin 2**	F'	CTGTGCCAGCAAGTTGACAT	59.04	50	113
			R'	TTCCTGAGCAGTTTGGCTCT	59.23	50	
**Mitophagy**	***Pink1***	**PTEN induced kinase 1**	F'	GTGGACCATCTGGTTCAGCA	59.96	55	75
			R'	TGAGTCCCACTCCACAAGGA	60.11	55	
	Prkn	**E3 ubiquitin-protein ligase parkin**	F'	AAGAAGACCACCAAGCCTTGTC	60.75	50	157
			R'	CAAACCAGTGATCTCCCATGC	59.25	52.38	
**Biogenesis**	***Pgc1a***	**Peroxisome Proliferator-Activated Receptor Gamma Coactivator-1 alpha**	F'	GTAAATCTGCGGGATGATGG	56.36	50	145
			R'	ATTGCTTCCGTCCACAAAA	55.62	42.11	
	Tfam	**Transcription Factor A, mitochondrial**	F'	TCCACAGAACAGCTACCCAA	58.57	50	84
			R'	CCACAGGGCTGCAATTTTCC	60.04	55	
